# Factors associated with safe and successful postoperative day 1 discharge after lung operations: a systematic review and meta-analysis

**DOI:** 10.1186/s13019-024-02505-4

**Published:** 2024-02-14

**Authors:** Russell Seth Martins, Asad Saulat Fatimi, Amna Irfan Ansari, Hamna Raheel, Kostantinos Poulikidis, M. Jawad Latif, Syed Shahzad Razi, Faiz Y. Bhora

**Affiliations:** 1 Division of Thoracic Surgery, Department of Surgery, Hackensack Meridian School of Medicine, Hackensack Meridian Health (HMH) Network–Central Region, 65 James Street, Edison, NJ 08820 USA; 2 Medical College, Aga Khan University Hospital, Karachi, 74800 Pakistan; 3 Dow Medical College, Dow University of Health Sciences, Karachi, 74200 Pakistan

**Keywords:** Lung operation, Segmentectomy, Wedge resection, Early discharge, Postoperative complications

## Abstract

**Background:**

A shorter length of stay (LOS) is associated with fewer hospital-acquired adverse conditions and decreased utilization of hospital resources. While modern perioperative care protocols have enabled some ambitious surgical teams to achieve discharge as early as within postoperative day 1 (POD1), most other teams remain cautious about such an approach due to the perceived risk of missing postoperative complications and increased readmission rates. We aimed to identify factors that would help guide surgical teams aiming for safe and successful POD1 discharge after lung resection.

**Methods:**

We searched the PubMed, Embase, Scopus, Web of Science and CENTRAL databases for articles comparing perioperative characteristics in patients discharged within POD1 (DWPOD1) and after POD1 (DAPOD1) following lung resection. Meta-analysis was performed using a random-effects model.

**Results:**

We included eight retrospective cohort studies with a total of 216,887 patients, of which 22,250 (10.3%) patients were DWPOD1. Our meta-analysis showed that younger patients, those without cardiovascular and respiratory comorbidities, and those with better preoperative pulmonary function are more likely to qualify for DWPOD1. Certain operative factors, such as a minimally invasive approach, shorter operations, and sublobar resections, also favor DWPOD1. DWPOD1 appears to be safe, with comparable 30-day mortality and readmission rates, and significantly less postoperative morbidity than DAPOD1.

**Conclusions:**

In select patients with a favorable preoperative profile, DWPOD1 after lung resection can be achieved successfully and without increased risk of adverse outcomes such as postoperative morbidity, mortality, or readmissions.

**Supplementary Information:**

The online version contains supplementary material available at 10.1186/s13019-024-02505-4.

## Introduction

Approximately 120,000 lung resections for lung cancer are performed annually in the United States [1]. Minimally invasive techniques such as video-assisted (VATS) and robotic-assisted (RATS) thoracoscopic surgery are increasingly becoming the standard of care, as they are associated with reduced complications, postoperative pain, and length of hospital stay (LOS) compared to open thoracotomy [2–5].

The average LOS after lung resection ranges from 4–5 days in the US. This is an improvement from the 7-day average LOS a decade ago, when open thoracotomy was more common [6]. A shorter LOS is associated with fewer hospital-acquired infections and decreased utilization of hospital resources [6], with approximately $1500 being saved per day reduction in LOS [7, 8]. To achieve earlier hospital discharges with optimal postoperative outcomes, healthcare systems in the US have developed various fast-track perioperative care protocols such as Enhanced Recovery after Surgery (ERAS®) [9, 10]. In thoracic surgery, these enhanced recovery protocols include recommendations for early removal of chest tubes, appropriate lung physiotherapy, and pain management to help effectuate earlier discharge [9, 11–13].

Measures such as these have enabled thoracic surgery teams in several institutions to achieve discharge as early as within the first postoperative day (POD1) without increasing readmissions [14–17]. However, most other teams remain cautious about such an approach due to the perceived risk of missing postoperative complications and increased readmission rates [16, 18]. To aim for successful and safe POD1 discharge in one’s practice, surgical teams must be able to recognize what patient characteristics may allow for a POD1 discharge. This study aims to identify factors that would help guide surgical teams aiming for successful POD1 discharge after lung resection.

## Methods

This meta-analysis adheres to the guidelines in the Cochrane Handbook for Systematic Reviews of Interventions and the Preferred Reporting Items for Systematic Reviews and Meta-Analysis (PRISMA) [19]. The completed PRISMA Checklist (2020) is available in Additional file 1: Section 1. An institutional review board approval was not required for this study as the data used is publicly available. This study was prospectively registered with PROSPERO (CRD42023406389).

### Search strategy

A comprehensive search of electronic databases including PubMed, Scopus, Embase, Web of Science, and Cochrane Central Register of Controlled Trials (CENTRAL), was conducted from inception to December 2022. Studies were also identified by manual searching and snowballing by reviewing bibliographies of relevant articles. Conference proceedings of published abstracts were also examined to identify grey literature. No restriction on time, language, study design, or sample size was placed. A detailed search strategy is mentioned in Additional file 1: Section 2.

### Study selection

Articles were shortlisted based on the following inclusion criteria:

1. Observational or interventional studies reporting original data.
2. Studies comparing data based on the following PICO (participants, intervention, comparator, outcome) parameters:
  **P**: Adult patients who underwent any type of lung resection (including anatomic and non-anatomic resections)
  **I**: Discharges within POD1 (DWPOD1)
  **C**: Discharges after POD1 (DAPOD1)
  **O**: Preoperative patient characteristics, intraoperative variables, or postoperative outcomes.

The exclusion criteria are as follows:

1. Studies including patients undergoing lung transplant.
2. Articles that failed to stratify outcomes based on groups of interest (DWPOD1 and DAPOD1).
3. Articles with no extractable or analyzable data based on POD1 discharge.
4. Articles published prior January 2000.

### Screening process and data extraction

All articles retrieved from the systematic search were exported to Endnote Reference Manager (Version X4; Clarivate Analytics, Philadelphia, Pennsylvania, USA) where duplicates were identified and removed. Two authors (HR and AIA) independently reviewed the articles initially on the bases of the title and abstract. Eventually, the full text of the shortlisted articles was read to ensure inclusion based on the eligibility criteria. A third author (ASF or RSM) was consulted in case of a discrepancy. General article data and perioperative variables were extracted from all short-listed articles by two independent reviewers (HR and AIA), with any conflict resolved by consensus with a third reviewer (ASF or RSM). Anatomic lung resection (AR) was defined by studies to include both lobectomies and segmentectomies.

In case of missing data in the article main text or Additional file 1, corresponding authors were contacted to retrieve the additional data for analysis. We also approximated means and standard deviations from medians and interquartile ranges where necessary, based on methodologies outlined by Wan et al. [20]. The continuous data that was approximated using these methods is shown in Additional file 1: Section 3.

Although two of the included studies, Drawbert et al. [14] and Mahenthiran et al. [21] performed propensity-score matched (PSM) analyses for postoperative mortality and readmission, only Drawbert et al. reported these data in a form suitable for meta-analysis. As such, no PSM data from Mahenthiran et al. could be incorporated into the meta-analysis. Moreover, for postoperative mortality and readmission, PSM data for Drawbert et al. was used instead of data from the unmatched cohort.

Two of the included studies, Mahenthiran et al. [21] and Patel et al. [18], had an overlapping patient cohort as they both used the American College of Surgeons National Surgical Quality Improvement Program (ACS-NSQIP) Database. For comparisons where both studies reported meta-analyzable data, we used the data from Patel et al. [18] because it included data from 2011 to 2019 (while Mahenthiran et al. only included data from 2011 to 2018).

As Towe et al. [22] stratified outcomes based on wedge resection (WR) and AR, data was extracted for the patients who underwent WR as only the WR group reported outcomes for patients DWPOD1. The remaining studies were comprised wholly of patient cohorts who underwent AR.

### Quality assessment and certainty of evidence assessment

Two authors (HR and AIA) independently assessed the risk of bias in all included articles using the risk of bias in non-randomized studies-of interventions (ROBINS-I) tool for observational studies [23]. The certainty of evidence for each outcome was independently determined using the GRADE (Grading of Recommendations, Assessment, Development, and Evaluations) approach [24] by two reviewers (HR and AIA) via the GRADE Pro Software (McMaster University and Evidence Prime Inc, Ontario, Canada). In case of any disagreements, a third reviewer was consulted (ASF). Publication bias was not included for any outcome given that the Cochrane Handbook advises not to generate funnel plots for outcomes with less than 10 studies [25], and our review quantitatively pooled 8 studies.

### Statistical analysis

All statistical analysis was conducted using RevMan (version 5.4; Copenhagen: The Nordic Cochrane Centre, The Cochrane Collaboration, 2022). Pooled results were represented as risk ratios (RRs) with 95% confidence intervals (CIs) for dichotomous outcome variables, and as odds ratios (ORs) with 95% CI for all other dichotomous variables. Continuous outcomes were presented as mean differences (MDs) with 95% CI. We used the Mantel–Haenszel random effects model to report the pooled RRs/ORs and the inverse variance random effects model to report the pooled MDs. Random effects meta-analysis models were also employed to offset the impact of variable sample sizes across the studies. This is because, given the shared between-study variance used in random-effects models, they lead to a more balanced distribution of weights despite differences in sample size [26]. It is also worth noting that while associations between DWPOD1 and continuous variables were statistically tested, the overall size of the MD may not necessarily be meaningful due to many continuous data being approximated from non-parametric measures. Forest plots were generated for all outcomes with greater than or equal to 3 studies. A *p* < 0.05 was considered statistically significant in all cases.

Heterogeneity due to between-study differences was assessed using Tau^2^, which quantifies between-study variability in effect sizes, and Higgins I^2^ statistics, which quantifies the proportion of variability in effect sizes that is attributable to heterogeneity (I^2^ = 0 was considered negligible, 1–50% was considered minimal, 50–75% moderate, and > 75% substantial). Additionally, sensitivity analyses were performed whereby the meta-analysis was re-conducted for each outcome by removing each study individually and evaluating its effect on the significance of the pooled result.

## Results

### Study characteristics

**Fig. 1:**
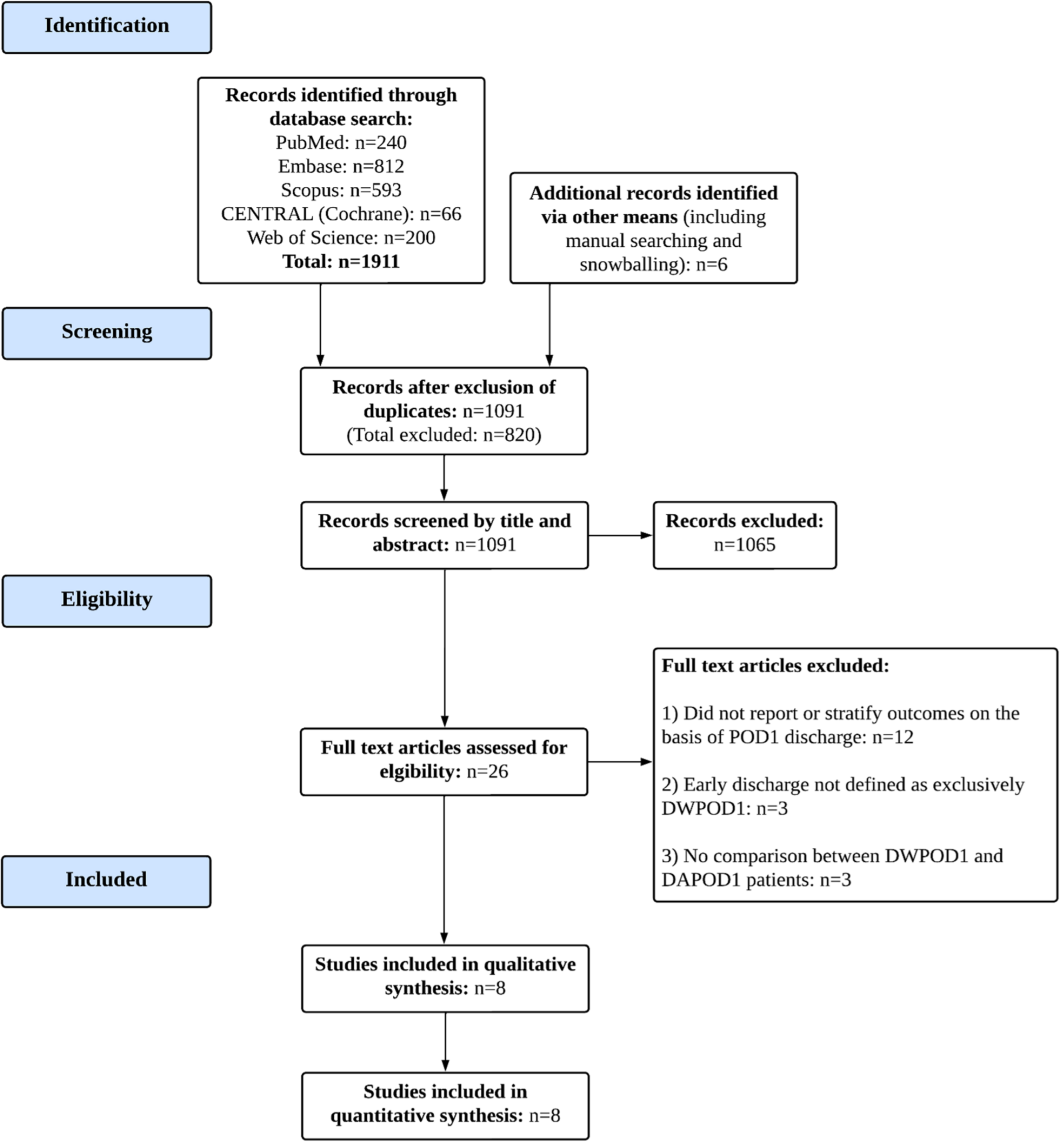
PRISMA Flowchart

We included eight studies [14–16, 18, 21, 22, 27, 28], all retrospective cohort studies, with a total of 216,887 patients (Fig. 1). Amongst these, 22,250 (10.3%) patients were discharged within POD1 (DWPOD1). All studies were conducted in the US and published in or after 2018. Six out of eight studies analyzed data from an existing multicentric database [14, 16, 18, 21, 27, 28], while the remaining two [15, 22] used institutional data. Two of the included studies performed propensity-score-matched analyses [14, 18]. All of the patients in Drawbert et al. [14], and the majority of patients in Greer et al. [27] (DWPOD1: 62.8%; DAPOD1: 64.7%) had Stage 1 malignancies, while the remaining studies did not provide information on TNM stage. Table 1 outlines relevant study characteristics of the articles included.

**Table 1:** Characteristics of included articles

First Author (Year)	DWPOD1—n (%)	Data Source	Minimally Invasive—n (%)		Malignant Diagnosis—n (%)		LOS in DAPOD1 (Days)
			DWPOD1	DAPOD1	DWPOD1	DAPOD1	
Drawbert et al. [14]	3879 (7.3) PSM: 3819 (50)	NCDB	1429 (36.8) PSM: 1400 (43.6)	20,246 (41.4) PSM: 1646 (44.3)	3879 (100) PSM: 3819 (100)	48,951 (100) PSM: 3819 (100)	Range = 2–7
Geraci et al. [15]	134 (52.9)	Institutional	134 (100)—all Robotic	119 (100)—all Robotic	123 (90.4)	102 (85.7)	> 1
Greer et al. [27]	150 (38.5)	STAR Database	150 (100)—all VATS	170 (70.83)—all VATS	145 (96.7)	235 (97.9)	Mean = 3.9
Linden et al. [16]	1821 (3.9)	STS-GTSD	1669 (91.7)	30,229 (67.9)	1821 (100)	44,504 (100)	Range = 2–9
Mahenthiran et al. [21]	1130 (7.8)	ACS-NSQIP	1130 (100)—all VATS	13,288 (100)—all VATS	971 (85.9)	12,195 (91.8)	Range = 2–29
Patel et al. [18]	854 (3.8) PSM: 788 (50)	ACS-NSQIP	770 (90.2)	10,617 (66.1)	854 (100)	16,604 (100)	Range = 2–20 +
Towe et al. [22]	448 (42.2)	Institutional	446 (99.6)	580 (94.6)	89 (19.9)	144 (23.5)	Range = 2–7
Tran et al. [28]	13,834 (16.4)	Nationwide Readmissions Database (NRD)	13,156 (95.1)	50,066 (71.2)	7664 (55.4)	46,480 (66.1)	Range = 2–5

*PSM:* Propensity Score Matched*; ACS-NSQIP*: American College of Surgeons National Surgical Quality Improvement Program; *NCDB*: National Cancer Database; *STAR*: Standardized Approach to Air Leak Reduction; *STS-GTSD*: Society of Thoracic Surgeons General Thoracic Surgery Database

### Demographics

All eight studies [14–16, 18, 21, 22, 27, 28] reported patient age and sex, while five studies [14, 18, 21, 22, 27] reported race. Meta-analysis revealed that younger age was associated with DWPOD1 (MD − 1.65 [95% CI − 2.56, − 0.75]; *p* < 0.001), while no significant associations were observed for sex and race (Additional file 1: Figs. 4.1–4.3**).**

### Primary diagnosis, comorbid conditions and functional status

**Fig. 2:**
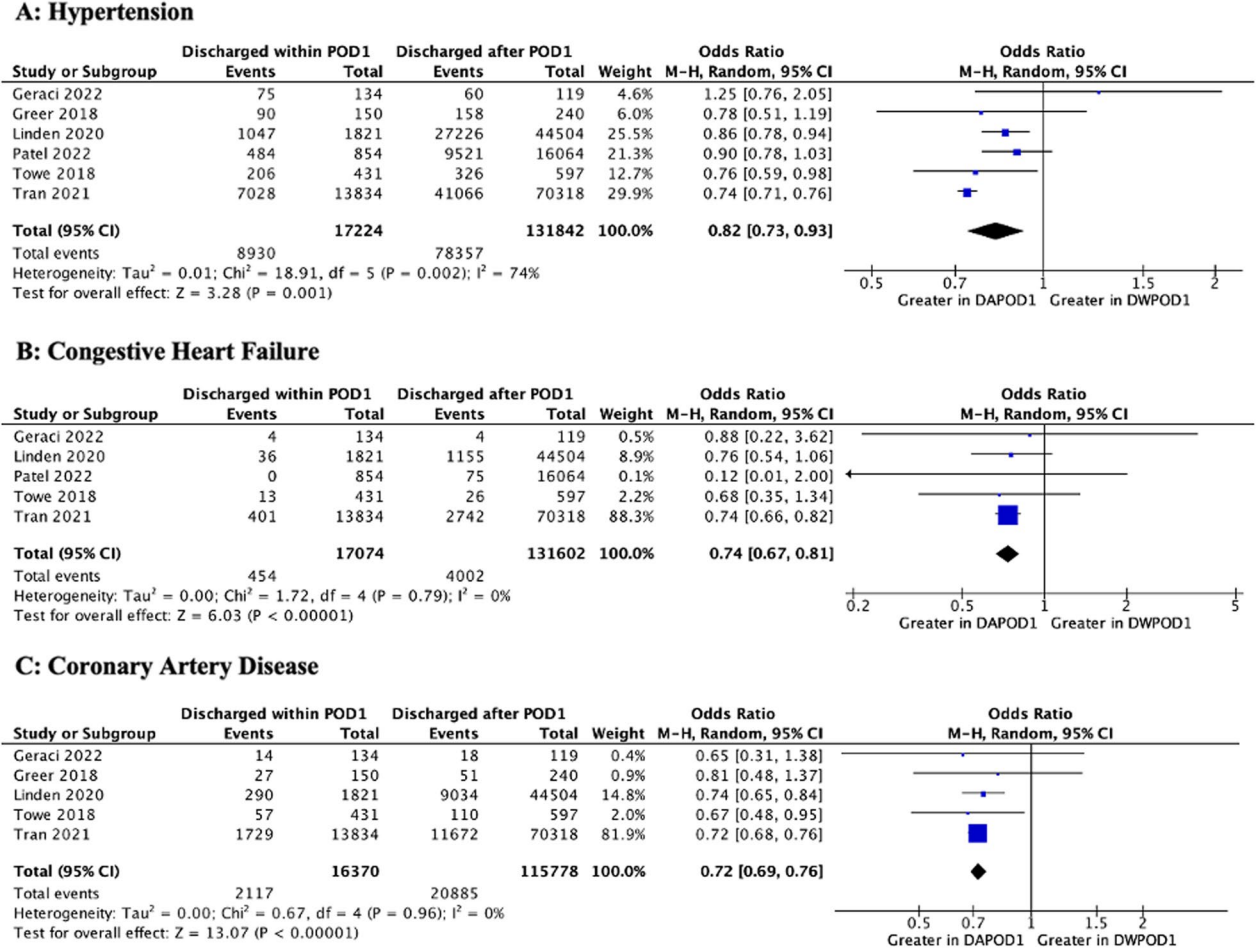
Meta-Analyses of Cardiovascular Comorbidities. M–H, Mantel–Haenszel; CI, confidence interval; df, degrees of freedom; P, probability value

**Fig. 3:**
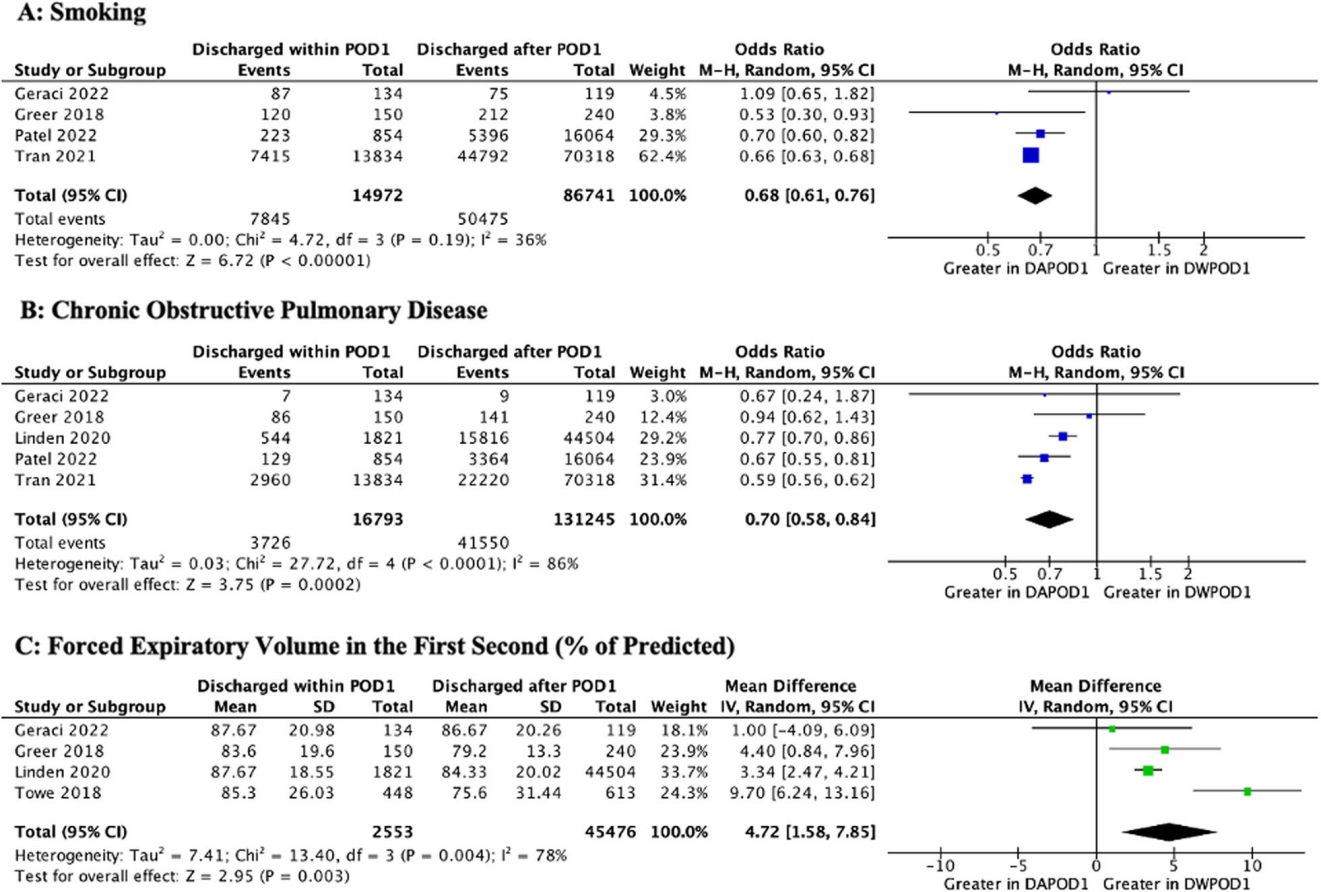
Meta-Analyses of Respiratory Comorbidities. M–H, Mantel–Haenszel; SD, standard deviation; IV, inverse variance; CI, confidence interval; df, degrees of freedom; P, probability value

A total of five studies [15, 21, 22, 27, 28] reported data separately for malignant and benign lung pathology. A diagnosis of lung cancer was associated with a significantly lower likelihood of DWPOD1 (OR 0.68 [0.56, 0.82]; *p* < 0.001). On analysis of comorbidities, a history of hypertension (HTN) (OR 0.82 [0.73, 0.93]; *p* = 0.001), smoking (OR 0.68 [0.61, 0.76]; *p* < 0.001), congestive heart failure (CHF) (OR 0.74 [0.67, 0.81]; *p* < 0.001), chronic obstructive pulmonary disease (COPD) (OR 0.70 [0.58, 0.84]; *p* < 0.001), coronary artery disease (CAD) (OR 0.72 [0.69, 0.76]; *p* < 0.001), or peripheral vascular disease (PVD) (OR 0.72 [0.56, 0.91]; *p* = 0.007) was associated with significantly lower DWPOD1 rates. While none of the studies reported multimorbidity (i.e., the presence of multiple comorbidities within the same patient), Drawbert et al. [14] (Charlson/Deyo Comorbidity Score) and Tran et al. [28] (Elixhauser Comorbidity Index) reported composite comorbidity scores. While these were non-meta-analyzable, both studies showed that DAPOD1 was associated with greater composite comorbidity scores at baseline. A higher BMI was also significantly associated with a greater likelihood of DWPOD1 (MD 0.44 [0.12, 0.75]; *p* = 0.007). With regards to pulmonary function, higher preoperative percentage of predicted forced expiratory volume in the first second (FEV_1_) (MD 4.72 [1.58, 7.85]; *p* = 0.002) and diffusing capacity of the lungs for carbon monoxide (DLCO) (MD 4.91 [1.73, 8.09]; *p* = 0.002) were associated with a higher likelihood of DWPOD1. ASA (American Society of Anesthesiology) Physical Status Classification, diabetes mellitus, and preoperative steroid medications were not associated with DWPOD1. These results are depicted in Figs. 2, 3, and Additional file 1: Figs. 4.4–4.10**.**

### Operative characteristics

**Fig. 4:**
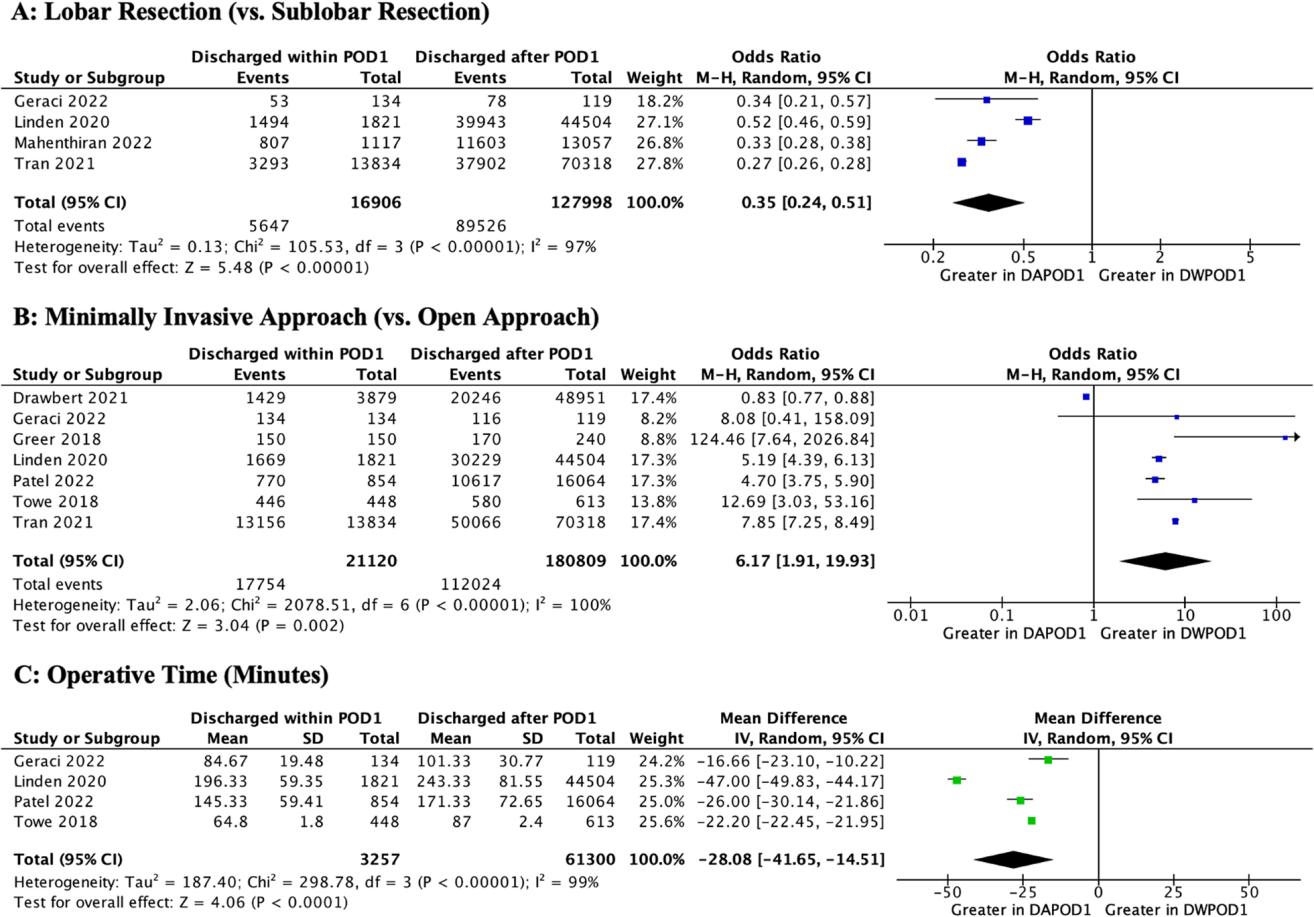
Meta-Analyses of Operative Characteristics. M–H, Mantel–Haenszel; SD, standard deviation; IV, inverse variance; CI, confidence interval; df, degrees of freedom; P, probability value

Location of the mass was not significantly associated with POD1 discharge. However, lobar resections were significantly associated with a lower likelihood of DWPOD1, compared to sub-lobar resections such as segmentectomies (OR 0.35 [0.24, 0.51]; *p* < 0.001). A minimally invasive approach was associated with significantly greater rates of DWPOD1, compared to open thoracotomy (OR 6.17 [1.91, 19.93]; *p* < 0.001). Shorter operations were also associated with a greater likelihood of DWPOD1 (MD − 28.08 [− 41.65, − 14.51]; *p* < 0.001). These results are depicted in Fig. 4**.**

### Postoperative characteristics

**Fig. 5:**
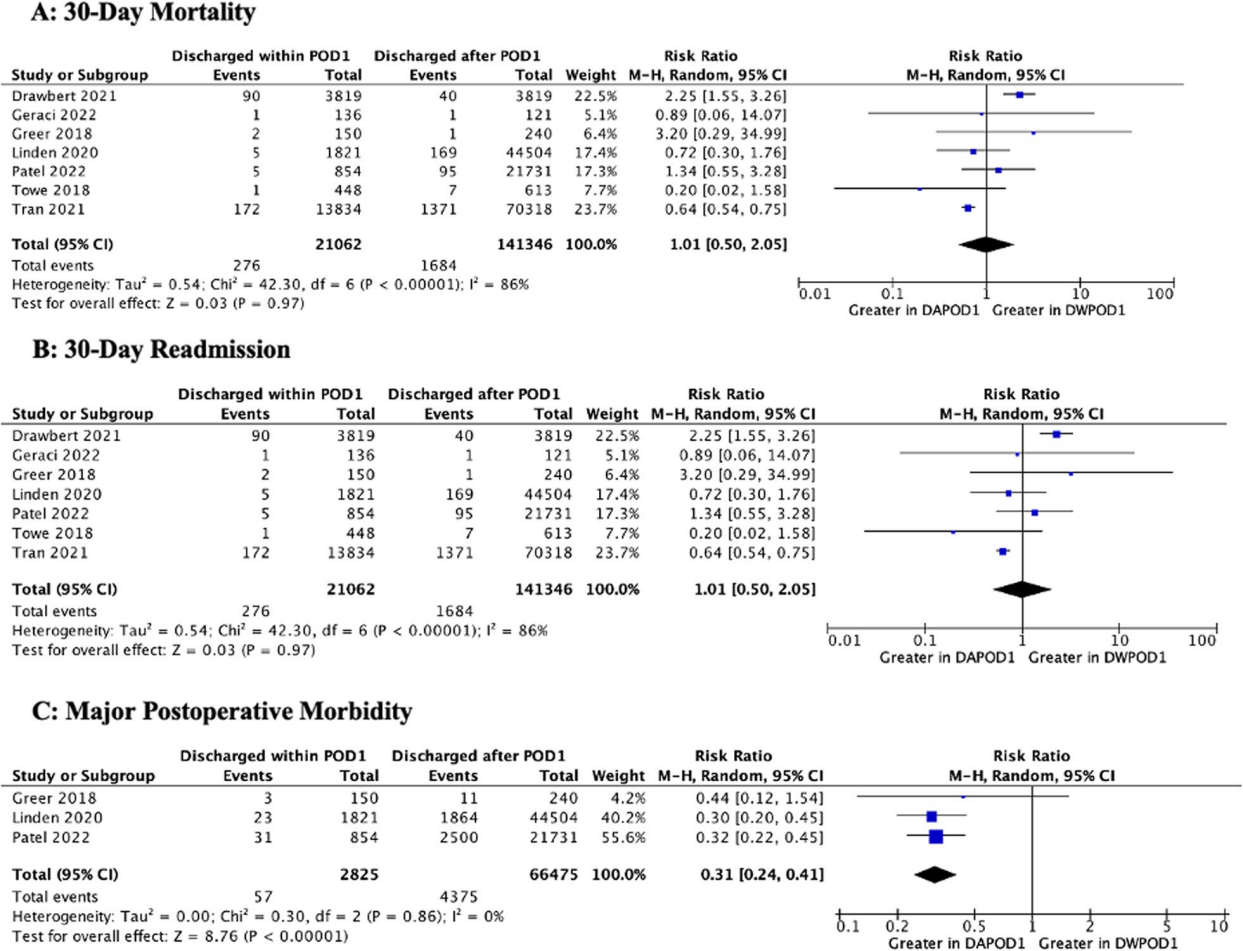
Meta-Analyses of Postoperative Characteristics. M–H, Mantel–Haenszel; SD, standard deviation; IV, inverse variance; CI, confidence interval; df, degrees of freedom; P, probability value

Chest tube management strategies were described in three articles [15, 22, 27], none of which considered chest tube removal to be an absolute criterion for hospital discharge. These have been summarized in Additional file 1: Table 5.1. Patients in the DWPOD1 were less likely to be discharged with a chest tube in place (OR 0.38 [0.15, 0.91]) and develop air leaks that persisted > 5 days (OR 0.19 [0.08–0.046]). There were no significant differences in 30-day mortality (RR: 1.01 [0.50, 2.05]) or 30-day readmission (RR 0.84 [0.62, 1.14]) between the DWPOD1 and DAPOD1 groups, although DWPOD1 patients were less likely to experience major postoperative morbidity (RR 0.31 [0.24, 0.41]; *p* < 0.001). However, sensitivity analysis revealed that on removal of Drawbert et al. [14] (which was the only study to report a significantly higher rate of 30-day mortality and 30-day readmission in the DWPOD1 group and also the only study with PSM data for these outcomes) DWPOD1 patients were less likely to experience mortality (RR: 0.70 [0.51, 0.96]) or readmission (RR: 0.75 [0.62, 0.89]). These results are depicted in Figs. 5 and Additional file 1: Figs. 4.11–4.12**.** When replacing the PSM data from Drawbert et al. with the data from the unmatched cohort, the pooled meta-analyzed result is insignificant for both outcomes (mortality: 1.04 [0.44, 2.42]; and readmission: 0.83 [0.59, 1.17]). A summary of all meta-analyses is shown in Table 2**.**

**Table 2:** Factors associated with DWPOD1 compared to DAPOD1 patients

Continuous variables					
Variable		Number of studies	MD [95% CI] for DWPOD1	*P*-value	GRADE certainty of evidence
Preoperative Characteristics	Age	7	− 1.65 [− 2.56, − 0.75]	< 0.001	Low
	BMI (kg/m^2^)	4	0.44 [0.12, 0.75]	0.007	High
	DLCO (% of Predicted)	4	4.91 [1.73, 8.09]	0.002	Moderate
	FEV1 (% of Predicted)	4	4.72 [1.58, 7.85]	0.003	Low
Operative Characteristics	Operation Duration (Minutes)	4	− 28.08 [− 41.65, − 14.51]	< 0.001	Very Low

Categorical variables					
Variable		Number of studies	RR/OR [95% CI] for DWPOD1	*P*-value	GRADE certainty of evidence
Preoperative Characteristics	Upper Lobe Pathology (vs. Lower/Middle Lobe)	4	1.04 [0.93, 1.16]	0.12	Low
	Smokers	4	0.68 [0.61, 0.76]	< 0.001	High
	White/Caucasian Race (vs. All Other Races)	4	1.02 [0.84, 1.24]	0.86	Moderate
	Preoperative Steroids	4	0.91 [0.62, 1.34]	0.63	Moderate
	PVD	4	0.72 [0.56, 0.91]	0.007	Moderate
	Malignant (vs. Benign) Lung Pathology	5	0.68 [0.56, 0.82]	< 0.001	High
	HTN	6	0.82 [0.73, 0.93]	0.001	Moderate
	Diabetes	5	0.96 [0.79, 1.16]	0.65	Moderate
	CAD	5	0.72 [0.69, 0.76]	< 0.001	High
	COPD	6	0.70 [0.58, 0.84]	< 0.001	Moderate
	CHF	5	0.74 [0.67, 0.81]	< 0.001	High
	Male Sex (vs. Female Sex)	7	1.03 [0.91, 1.17]	0.60	Low
	ASA Score < 3 (vs. ASA Score ≥ 3)	4	2.29 [0.67, 7.82]	0.18	Very Low
Operative Characteristics	Minimally Invasive Approach (vs. Open Approach)	7	6.17 [1.91, 19.93]	0.002	Moderate
	Lobar Resection (vs. Sublobar Resection)	4	0.35 [0.24, 0.51]	< 0.001	High
Postoperative Characteristics	Postoperative 30-Day Readmission	7	0.84 [0.62, 1.14]	0.27	Low
	Postoperative 30-Day Mortality	7	1.01 [0.50, 2.05]	0.97	Low
	Major Morbidity	3	0.31 [0.24, 0.41]	< 0.001	High
	Discharged With Chest Tube	3	0.38 [0.15, 0.91]	0.03	Moderate
	Air Leak (> 5 Days)	2	0.19 [0.08, 0.46]	< 0.001	High

DWPOD1: Discharged Within Postoperative Day 1; DAPOD1: Discharged After Postoperative Day 1; OR: Odds Ratio; RR: Risk Ratio; MD: Mean Difference; CI: Confidence Interval; FEV1: Forced Expiratory Volume In 1 Second; DLCO: Diffusing Capacity of the Lung for Carbon Monoxide

### Heterogeneity

Substantial effect size variation attributable to inter-study heterogeneity was observed for the meta-analysis of age, HTN, COPD, FEV_1_, DLCO, type of resection, duration of operation, operative approach, and major postoperative morbidity. Moderate effect size variation attributable to inter-study heterogeneity was observed for the meta-analysis of nature of pathology and PVD. Minimal or low effect size variation attributable to inter-study heterogeneity was observed for the meta-analysis of CHD, CAD, BMI, and smoking history (Additional file 1: Section 6).

### Additional sensitivity analyses

The relatively larger sample size of Tran et al. [28] accounted for its larger weight in the outcomes where it was pooled. As such, a sensitivity analysis was conducted to investigate its impact on these outcomes and to reduce bias. There was no change in the significance of the meta-analyzed result upon removal of Tran et al. [28] for any of the variables barring nature of the lung pathology (malignant vs. benign), where removing the study resulted in a non-significant result (OR 0.78 [0.51, 1.21]).

In addition, given that Towe et al. [22] was the only study which had data from patients who underwent WR, a sensitivity analysis was conducted to determine whether similar results would be obtained when pooling studies with purely AR patients. There was no change in the significance of the pooled result upon removal of Towe et al. in any of the variables except for race where removing the study resulted in a significant result (OR 0.92 [0.85, 1.00]; *p* = 0.04).

Additional details regarding these sensitivity analyses are present in Additional file 1: Section 7.

### Risk of bias assessment (ROBINS-I)

5 [14–16, 21, 28] out of the 8 included studies were deemed to have an overall low risk of bias as per the ROBINS-I tool, with the remaining 3 [18, 22, 27] having a moderate risk of bias. The domain-wise results of the quality assessment are present in the Additional file 1: Section 8.

### Certainty of evidence

The overall certainty of evidence was low to moderate. Of the 25 meta-analyzed comparisons, 8 were deemed to have a high certainty of evidence, 9 were deemed to have a moderate certainty of evidence, 6 were deemed to have a low certainty of evidence, and 2 were deemed to have a very low quality of evidence. The domain-wise GRADE evidence profile is available in Additional file 1: Section 9.

## Discussion

In this meta-analysis, we aimed to identify factors associated with successful DWPOD1 after lung resection, both anatomic and non-anatomic, and evaluate outcomes after DWPOD1. Our results show that younger patients, those without cardiovascular and respiratory comorbids (HTN, CHF, PVD, CAD, COPD, smoking history), and better preoperative pulmonary function (FEV_1_ and DLCO) are more likely to qualify for DWPOD1. Interestingly, a higher BMI was found to favor DWPOD1. Certain operative factors, such as a minimally invasive approach, shorter operations, and sublobar resections, also favor DWPOD1. Lastly, DWPOD1 appears to be safe when implemented in a favorable patient cohort, with comparable 30-day mortality and readmission rates, and significantly less postoperative morbidity.

The patient characteristics that favor DWPOD1 likely do so by streamlining postoperative recovery after lung resection. Younger patients have better post-anesthesia cognitive recovery and are less frail, allowing for earlier and safer postoperative mobilization [29, 30]. Earlier ambulation facilitates DWPOD1 by improving pain and cardiorespiratory function. Moreover, older patients undergoing lung operations may also be more likely to require discharge to a non-home care or rehabilitation institution [31], the logistics of which may lead to a longer hospital LOS. Interestingly, higher BMI may favor quicker postoperative recovery—the so called “obesity paradox”—though mechanisms underlying this association remain unclear and require further exploration [32]. Our results provide thoracic surgery teams with a yardstick to assess overall suitability for deliberately expedited postoperative care where the team can be confident about a safe and successful DWPOD1. More research is needed to develop appropriate risk stratification systems that consider these key patient characteristics and allow surgeons to determine suitability for DWPOD1 in an objective and standardized manner. A preliminary attempt in this regard has been presented by Towe et al. [33], with their risk score being based on a multivariable regression model with constituent variables like those identified as significant in our study (patient age, BMI, CAD, COPD, operative approach, and duration of operation). Our results may help guide future iterations of such risk calculators.

Perhaps most importantly in the context of lung resection, preoperative pulmonary health and function appeared to be a strong predictor of successful DWPOD1. Lung resections may be associated with significant reductions in pulmonary function (on average about 22% reduction after lobectomy) [34]. It is thus no surprise that evidence-based clinical practice guidelines (EBCPGs) by the ERAS ® Society and the European Society of Thoracic Surgeons (ESTS) place heavy emphasis on maximizing pulmonary function [35]. A proactive approach to pulmonary prehabilitation, particularly in patients with decreased lung function, can help reduce hospital LOS and should potentially be considered as a routine component of care in ambitious surgical teams aiming for DWPOD1 [35].

In addition, chest tube management is a key component of an accelerated discharge program. None of the three studies describing chest tube removal strategies [15, 22, 27] considered tube removal or absence of an air leak to be absolute criteria for hospital discharge. While we concur with this general philosophy, there are other factors that must also be considered. The extent of resection and surgical approach may be important determinants of successful postoperative outpatient chest tube management. One Chinese study of 95 patients from 2020 suggested that outpatient chest tube management can be successfully achieved in select patients who undergo minimally invasive segmentectomies [36]. However, another study of 253 patients from 2022 suggested that discharge with a chest tube, irrespective of resection type, surgical approach, and functional status, may be associated with serious adverse outcomes including need for reoperation and empyema [37]. Moreover, patients and families must be provided with in-depth education regarding chest tube care at home, potential complications, and must be provided with an efficient and reliable means to contact the thoracic surgery team in case of concerns. In practices where the first routine outpatient follow-up after discharge occurs after approximately 5 days postoperatively, it may, therefore, be more prudent to wait an additional day (till POD2) for inpatient chest tube removal in a patient with a minor air leak/high chest tube output. The alternative is sending the patient with an indwelling tube and either scheduling an additional early visit for tube removal or waiting until the POD5 follow-up visit to do so—both cases pose an additional inconvenience to the patient.

We believe that DWPOD1 can be considered safe in patients with favorable preoperative baseline characteristics. There were no significant differences in 30-day mortality or 30-day readmission between the DWPOD1 and DAPOD1 groups, and DWPOD1 patients were less likely to experience major postoperative morbidity. Drawbert et al. [14] was the only study to report significantly worse postoperative outcomes amongst the DWPOD1 group, with this association seen only in low- and medium-volume centers. Interestingly, exclusion of Drawbert et al. from the meta-analysis resulted in DWPOD1 having significantly lower rates of 30-day mortality and 30-day readmission.

Lastly, we also believe that the decision to discharge a patient early should consider each individual patient’s personal circumstances. Elderly patients may have inadequate support structures at home and thus require skilled aid to assist them during the early postoperative period or may be discharged to a non-home facility. Both of these scenarios may be associated with additional costs, and thus the cost savings from hospital LOS reduction must be weighed against potential resultant out-of-hospital costs.

This study is not without its limitations. The meta-analysis for most variables demonstrated moderate-to-substantial heterogeneity, with this likely being a function of differences in patient case-mix and differences in data sources. Additionally, as several of the datasets used by the included articles were national datasets, there is the possibility of overlapping patient data between studies. The use of large databases also meant that the data in these articles were retrospective and non-granular. Only one study presented data for patients undergoing WR, precluding any pooled subgroup analysis exploring factors associated with DWPOD1 in a WR cohort exclusively. In addition, several key variables were not reported by the studies which is a known shortcoming of retrospective database studies [38]. These include patient frailty, diagnosis-related data (stage of malignancy), data on important postoperative in-patient milestones after lung operations (intravenous fluid administration, use of opiate analgesia and postoperative nausea and vomiting therapy, feeding, mobilization, and timing of chest drain and catheter removal) [39]. In addition, the type of thoracotomy (postero-lateral i.e., conventional or muscle-sparing) was not specified in most studies. Moreover, as DWPOD1 currently seems to be a routine practice only amongst select surgical teams across the US, it is also worthwhile to explore surgeon-level practices, workflow protocols, and staffing models that enable expedited discharges. The impact of hospital-level factors, such as hospital operative volume, should also be evaluated, as there is evidence of a volume-outcome relationship for lung operations [40, 41].

## Conclusion

Factors promoting DWPOD1 after lung resection include favorable preoperative patient characteristics, notably cardiovascular and pulmonary healthy function, and operative factors, such as a minimally invasive approach and shorter operations. In select patients, DWPOD1 can be achieved safely and successfully, without increased risk of postoperative morbidity, mortality, or readmissions. However, there is no single element that can predict whether patients are suitable for DWPOD1. The complex interplay of several preoperative and intraoperative factors necessitates the development of appropriate risk calculators that considers key patient characteristics to determine suitability for DWPOD1. Future research must investigate additional key variables in the continuum of perioperative care, including postoperative in-patient milestones, surgeon factors, and workflow protocols.

### Supplementary Information

**Additional file 1**. Supplementary Material.

## Data Availability

The data that support the findings of this study were sourced directly from the published studies included in this systematic review and meta-analysis.

## Abbreviations

VATS: Video-assisted thoracoscopic surgery
RATS: Robotic-assisted thoracoscopic surgery
LOS: Length of hospital stay
ERAS: Enhanced recovery after surgery
POD1: First postoperative day
PRISMA: Preferred reporting items for systematic reviews and meta-analysis
CENTRAL: Cochrane central register of controlled trials
DWPOD1: Discharges within POD1
DAPOD1: Discharges after POD1
ACS-NSQIP: American college of surgeons national surgical quality improvement program
ROBINS-I: Risk of bias in non-randomized studies-of interventions
GRADE: Grading of recommendations, assessment, development, and evaluations
PSM: Propensity-score-matched
RR: Risk ratio
OR: Odds ratio
MD: Mean difference
CI: Confidence interval
HTN: Hypertension
CHF: Congestive heart failure
COPD: Chronic obstructive pulmonary disease
CAD: Coronary artery disease
PVD: Peripheral vascular disease
FEV_1_: Forced expiratory volume in the first second
DLCO: Diffusing capacity of the lungs for carbon monoxide
ASA: American society of anesthesiologists
EBCPGs: Evidence-based clinical practice guidelines
ESTS: European society of thoracic surgeons
TNM: Tumor extent, nodal spread, presence of metastasis
