# Supplementary material for: Factors associated with safe and successful postoperative day 1 discharge after lung operations: a systematic review and meta-analysis
Source: J Cardiothorac Surg. 2024 Feb 14;19:91. doi: 10.1186/s13019-024-02505-4 (PMC10865531; doi:10.1186/s13019-024-02505-4)
Supplement: Supplementary file 1 — Additional file 1. Supplementary Material. [file 13019_2024_2505_MOESM1_ESM.docx]

**Factors associated with Safe and Successful Postoperative Day 1 Discharge after Lung Operations:**

**A Systematic Review and Meta-Analysis**

**Supplementary Material**

*Supplementary Section 1:* PRISMA Checklist (2020)

*Supplementary Section 2:* Complete Search Strings

*Supplementary Section 3:* Estimations from Non-Parametrically Distributed Data

*Supplementary Section 4:* Forest Plots (not included in the main manuscript)

*Supplementary Section 5:* Chest Tube Removal Strategies

*Supplementary Section 6:* Heterogeneity of Significant Outcomes

*Supplementary Section 7:* Sensitivity Analyses

*Supplementary Section 8:* Study Quality Assessment

*Supplementary Section 9:* GRADE Evidence Profile

**Section 1: PRISMA Checklist (2020)**

| **Section and Topic** | **Item #** | **Checklist item** | **Location where item is reported** |
| --- | --- | --- | --- |
| **TITLE** | | |  |
| Title | 1 | Identify the report as a systematic review. | Page 1 |
| **ABSTRACT** | | |  |
| Abstract | 2 | See the PRISMA 2020 for Abstracts checklist. | Page 2 |
| **INTRODUCTION** | | |  |
| Rationale | 3 | Describe the rationale for the review in the context of existing knowledge. | Page 4 |
| Objectives | 4 | Provide an explicit statement of the objective(s) or question(s) the review addresses. | Page 4 |
| **METHODS** | | |  |
| Eligibility criteria | 5 | Specify the inclusion and exclusion criteria for the review and how studies were grouped for the syntheses. | Page 5 |
| Information sources | 6 | Specify all databases, registers, websites, organisations, reference lists and other sources searched or consulted to identify studies. Specify the date when each source was last searched or consulted. | Page 5 |
| Search strategy | 7 | Present the full search strategies for all databases, registers and websites, including any filters and limits used. | Supplementary Section 2 |
| Selection process | 8 | Specify the methods used to decide whether a study met the inclusion criteria of the review, including how many reviewers screened each record and each report retrieved, whether they worked independently, and if applicable, details of automation tools used in the process. | Page 6 |
| Data collection process | 9 | Specify the methods used to collect data from reports, including how many reviewers collected data from each report, whether they worked independently, any processes for obtaining or confirming data from study investigators, and if applicable, details of automation tools used in the process. | Page 6 |
| Data items | 10a | List and define all outcomes for which data were sought. Specify whether all results that were compatible with each outcome domain in each study were sought (e.g. for all measures, time points, analyses), and if not, the methods used to decide which results to collect. | Page 6 |
|  | 10b | List and define all other variables for which data were sought (e.g. participant and intervention characteristics, funding sources). Describe any assumptions made about any missing or unclear information. | Page 6 |
| Study risk of bias assessment | 11 | Specify the methods used to assess risk of bias in the included studies, including details of the tool(s) used, how many reviewers assessed each study and whether they worked independently, and if applicable, details of automation tools used in the process. | Page 6 |
| Effect measures | 12 | Specify for each outcome the effect measure(s) (e.g. risk ratio, mean difference) used in the synthesis or presentation of results. | Page 7 |
| Synthesis methods | 13a | Describe the processes used to decide which studies were eligible for each synthesis (e.g. tabulating the study intervention characteristics and comparing against the planned groups for each synthesis (item #5)). | Page 6 |
|  | 13b | Describe any methods required to prepare the data for presentation or synthesis, such as handling of missing summary statistics, or data conversions. | Page 7 |
|  | 13c | Describe any methods used to tabulate or visually display results of individual studies and syntheses. | Page 7 |
|  | 13d | Describe any methods used to synthesize results and provide a rationale for the choice(s). If meta-analysis was performed, describe the model(s), method(s) to identify the presence and extent of statistical heterogeneity, and software package(s) used. | Page 7 |
|  | 13e | Describe any methods used to explore possible causes of heterogeneity among study results (e.g. subgroup analysis, meta-regression). | Page 7 |
|  | 13f | Describe any sensitivity analyses conducted to assess robustness of the synthesized results. | Page 7 |
| Reporting bias assessment | 14 | Describe any methods used to assess risk of bias due to missing results in a synthesis (arising from reporting biases). | Page 6 |
| Certainty assessment | 15 | Describe any methods used to assess certainty (or confidence) in the body of evidence for an outcome. | Page 6 |
| **RESULTS** | | |  |
| Study selection | 16a | Describe the results of the search and selection process, from the number of records identified in the search to the number of studies included in the review, ideally using a flow diagram. | Page 8/Table 1 |
|  | 16b | Cite studies that might appear to meet the inclusion criteria, but which were excluded, and explain why they were excluded. | Figure 1 |
| Study characteristics | 17 | Cite each included study and present its characteristics. | Table 1 |
| Risk of bias in studies | 18 | Present assessments of risk of bias for each included study. | Supplementary Section 7 |
| Results of individual studies | 19 | For all outcomes, present, for each study: (a) summary statistics for each group (where appropriate) and (b) an effect estimate and its precision (e.g. confidence/credible interval), ideally using structured tables or plots. | Page 8, 9, 10, Table 2 |
| Results of syntheses | 20a | For each synthesis, briefly summarise the characteristics and risk of bias among contributing studies. | Supplementary Section 7 |
|  | 20b | Present results of all statistical syntheses conducted. If meta-analysis was done, present for each the summary estimate and its precision (e.g. confidence/credible interval) and measures of statistical heterogeneity. If comparing groups, describe the direction of the effect. | Page 8, 9, 10, Table 2 |
|  | 20c | Present results of all investigations of possible causes of heterogeneity among study results. | Page 9, 10, Supplementary Section 6 |
|  | 20d | Present results of all sensitivity analyses conducted to assess the robustness of the synthesized results. | Page 9 |
| Reporting biases | 21 | Present assessments of risk of bias due to missing results (arising from reporting biases) for each synthesis assessed. | Supplementary Section 7 |
| Certainty of evidence | 22 | Present assessments of certainty (or confidence) in the body of evidence for each outcome assessed. | Page 10, Supplementary Section 8 |
| **DISCUSSION** | | |  |
| Discussion | 23a | Provide a general interpretation of the results in the context of other evidence. | Page 11, 12, 13 |
|  | 23b | Discuss any limitations of the evidence included in the review. | Page 12 |
|  | 23c | Discuss any limitations of the review processes used. | Page 12 |
|  | 23d | Discuss implications of the results for practice, policy, and future research. | Page 12, 13 |
| **OTHER INFORMATION** | | |  |
| Registration and protocol | 24a | Provide registration information for the review, including register name and registration number, or state that the review was not registered. | Page 5 |
|  | 24b | Indicate where the review protocol can be accessed, or state that a protocol was not prepared. | Page 5 |
|  | 24c | Describe and explain any amendments to information provided at registration or in the protocol. | N/A |
| Support | 25 | Describe sources of financial or non-financial support for the review, and the role of the funders or sponsors in the review. | Page 14 |
| Competing interests | 26 | Declare any competing interests of review authors. | Page 14 |
| Availability of data, code and other materials | 27 | Report which of the following are publicly available and where they can be found: template data collection forms; data extracted from included studies; data used for all analyses; analytic code; any other materials used in the review. | Page 14 |

*From:*  Page MJ, McKenzie JE, Bossuyt PM, Boutron I, Hoffmann TC, Mulrow CD, et al. The PRISMA 2020 statement: an updated guideline for reporting systematic reviews. BMJ 2021;372:n71. doi: 10.1136/bmj.n71

For more information, visit: <http://www.prisma-statement.org/>

**Section 2: Search Strings** *(searched in November 2022)*

**PubMed: 240 Articles**

("Postoperative day 1"[All Fields] OR "POD1"[All Fields] OR "first postoperative day"[All Fields] OR "early"[All Fields] OR "day 1"[All Fields] OR "day one"[All Fields]) AND "discharge"[All Fields] AND ("lung resection"[All Fields] OR "anatomic lung resection"[All Fields] OR "pulmonary resection"[All Fields] OR "lobectomy"[All Fields] OR "segmentectomy"[All Fields] OR "pneumonectomy"[All Fields] OR "pneumonectomy"[MeSH Terms] OR "Endoscopic Lung Volume Reduction"[All Fields] OR "Bronchoscopic Lung Volume Reduction"[All Fields] OR "Lung Volume Reduction"[All Fields] OR "Lung Volume Reduction Surgery"[All Fields] OR "wedge resection"[All Fields] OR "pulmonary neoplas*"[All Fields] OR "lung neoplas*"[All Fields] OR "lung neoplasms"[MeSH Terms] OR "lung cancer"[All Fields] OR "pulmonary cancer"[All Fields])

**Scopus: 593 Articles**

TITLE-ABS-KEY ( ( ( "Postoperative day 1"  OR  "POD1"  OR  "first postoperative day"  OR  "early"  OR  "day 1"  OR  "day one" )  AND  "discharge" )  AND  ( "lung resection"  OR  "anatomic lung resection"  OR  "pulmonary resection"  OR  "lobectomy"  OR  "segmentectomy"  OR  "pneumonectomy"  OR  "Endoscopic Lung Volume Reduction"  OR  "Bronchoscopic Lung Volume Reduction"  OR  "Lung Volume Reduction"  OR  "Lung Volume Reduction Surgery"  OR  "wedge resection"  OR  "pulmonary neoplas*"  OR  "lung neoplas*"  OR  "lung cancer"  OR  "pulmonary cancer" ) )

**Embase: 812 Articles**

(**'postoperative day 1'** OR **'pod1'** OR **'first postoperative day'** OR **'early'** OR **'day 1'** OR **'day one'**) AND **'discharge'** AND (**'lung resection'** OR **'anatomic lung resection'** OR **'pulmonary resection'** OR **'lobectomy'** OR **'segmentectomy'** OR **'pneumonectomy'** OR **'endoscopic lung volume reduction'** OR **'bronchoscopic lung volume reduction'** OR **'lung volume reduction'** OR **'lung volume reduction surgery'** OR **'wedge resection'** OR **'pulmonary neoplas*'** OR **'lung neoplas*'** OR **'lung cancer'** OR **'pulmonary cancer'**)

**Web of Science: 200 Articles**

((“Postoperative day 1” OR “POD1” OR “first postoperative day” OR “early” OR “day 1” OR “day one”) AND “discharge”) AND (“lung resection” OR “anatomic lung resection” OR “pulmonary resection” OR “lobectomy” OR “segmentectomy” OR “pneumonectomy” OR “Endoscopic Lung Volume Reduction” OR “Bronchoscopic Lung Volume Reduction” OR “Lung Volume Reduction” OR “Lung Volume Reduction Surgery” OR “wedge resection” OR “pulmonary neoplas*” OR “lung neoplas*” OR “lung cancer” OR “pulmonary cancer”)

**Cochrane: 66 Articles**

ID Search Hits

#1 “Postoperative day 1” OR “POD1” OR “first postoperative day” OR “early” OR “day 1” OR “day one” 171419

#2 "discharge" 41509

#3 “lung resection” OR “anatomic lung resection” OR “pulmonary resection” OR “lobectomy” OR “segmentectomy” OR “pneumonectomy” OR “Endoscopic Lung Volume Reduction” OR “Bronchoscopic Lung Volume Reduction” OR “Lung Volume Reduction” OR “Lung Volume Reduction Surgery” OR “wedge resection” OR “pulmonary neoplas*” OR “lung neoplas*” OR “lung cancer” OR “pulmonary cancer” 24985

#4 (#1 AND #2) AND #3 102

**Section 3: Estimations from Non-Parametrically Distributed Data**

**Age**

**Means and SD’s that have been calculated from Medians, Ranges, and/or IQR’s*

***Multiple means and standard deviations were combined to give a composite mean and SD*

| **Study** | **Sample Size** | | **POD1** | | **Non-POD1** | |
| --- | --- | --- | --- | --- | --- | --- |
|  | **POD1** | **Non-POD1** | **Mean** | **SD** | **Mean** | **SD** |
| Drawbert et al. 2022* | 3879 | 48951 | 67.33 | 5.93 | 67.67 | 9.64 |
| Geraci et al. 2022* | 134 | 119 | 67.67 | 9.74 | 68.67 | 15.01 |
| Greer et al. 2018 | 150 | 240 | 65 | 10 | 67 | 11 |
| Linden et al. 2020* | 1821 | 44504 | 66 | 10.39 | 67.67 | 9.64 |
| Mahenthiran et al. 2022 | 1130 | 13288 | 54.9 | 10.7 | 67.21** | 9.94** |
| Patel et al. 2022* | 854 | 16064 | 66.67 | 9.65 | 67.67 | 9.64 |
| Towe et al. 2018 | 448 | 613 | 62.3 | 0.62 | 64.8 | 0.6 |
| Tran et al. 2021 | 13834 | 70318 | 62.6 | 12.1 | 65.3 | 11.1 |

**BMI**

**Means and SD’s that have been calculated from Medians, Ranges, and/or IQR’s*

| **Study** | **Sample Size** | | **POD1** | | **Non-POD1** | |
| --- | --- | --- | --- | --- | --- | --- |
|  | **POD1** | **Non-POD1** | **Mean** | **SD** | **Mean** | **SD** |
| Geraci et al. 2022* | 134 | 119 | 27.33 | 7.49 | 25.33 | 5.25 |
| Greer et al. 2018 | 150 | 240 | 27.8 | 5.6 | 27.5 | 5.6 |
| Linden et al. 2020* | 1821 | 44504 | 27.63 | 5.42 | 27.17 | 5.41 |
| Patel et al. 2022* | 854 | 16064 | 28 | 5.20 | 27.73 | 5.41 |

**FEV1 (% of Predicted)**

**Means and SD’s that have been calculated from Medians, Ranges, and/or IQR’s*

***SD has been computed from Standard Error*

| **Study** | **Sample Size** | | **POD1** | | **Non-POD1** | |
| --- | --- | --- | --- | --- | --- | --- |
|  | **POD1** | **Non-POD1** | **Mean** | **SD** | **Mean** | **SD** |
| Geraci et al. 2022* | 134 | 119 | 87.67 | 20.98 | 86.67 | 20.26 |
| Greer et al. 2018 | 150 | 240 | 83.6 | 19.6 | 79.2 | 13.3 |
| Linden et al. 2020* | 1821 | 44504 | 87.67 | 18.55 | 84.33 | 20.02 |
| Towe et al. 2018** | 448 | 613 | 85.3 | 26.03 | 75.6 | 31.44 |

**Diffusing Capacity of the Lung for Carbon Monoxide (DLCO) (% of Predicted)**

**Means and SD’s that have been calculated from Medians, Ranges, and/or IQR’s*

***SD has been computed from Standard Error*

| **Study** | **Sample Size** | | **POD1** | | **Non-POD1** | |
| --- | --- | --- | --- | --- | --- | --- |
|  | **POD1** | **Non-POD1** | **Mean** | **SD** | **Mean** | **SD** |
| Geraci et al. 2022* | 134 | 119 | 83 | 18.73 | 79 | 18.01 |
| Greer et al. 2018 | 150 | 240 | 77.5 | 18.3 | 75.6 | 19.1 |
| Linden et al. 2020* | 1821 | 44504 | 77.67 | 20.03 | 74 | 20.76 |
| Towe et al. 2018** | 448 | 613 | 83.9 | 32.81 | 73.2 | 32.43 |

**Section 4: Forest Plots (not included in the main manuscript)**

**Legend:** M-H, Mantel-Haenszel; SD, standard deviation; IV, inverse variance; CI, confidence interval; df, degrees of freedom; P, probability value

**Supplementary Figure 4.1: Age**

**
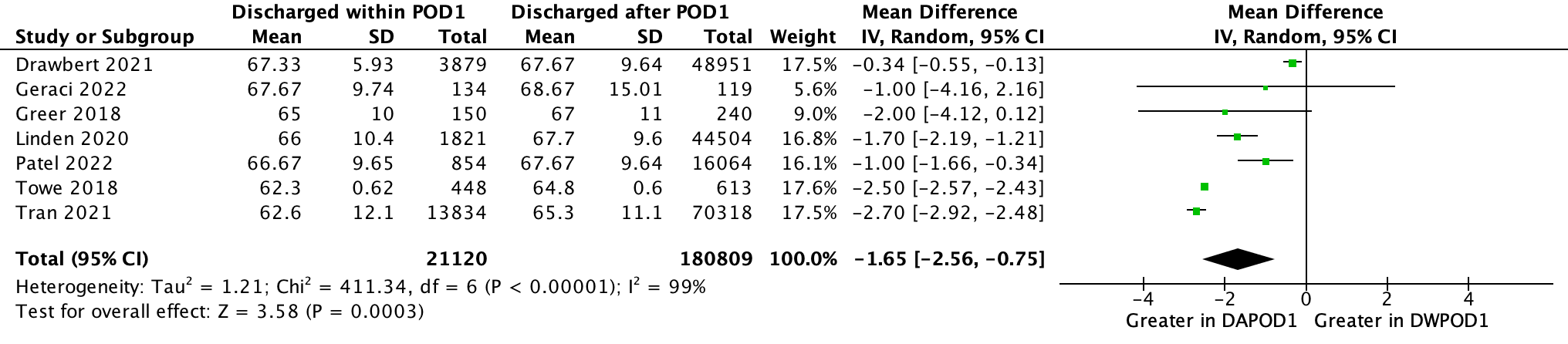
**

**Supplementary Figure 4.2: Male Sex (vs. Female Sex)**

**
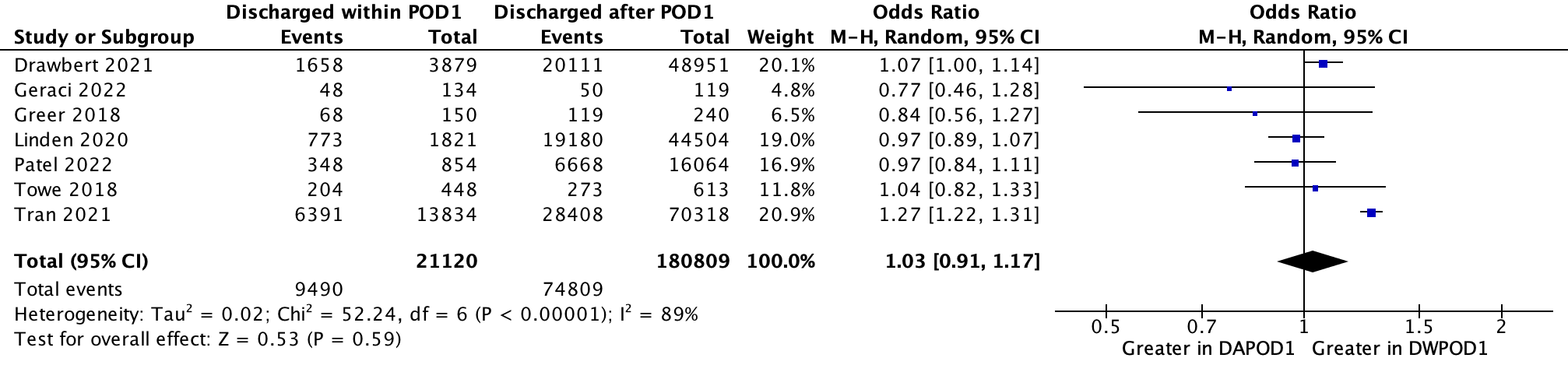
**

**Supplementary Figure 4.3: White/Caucasian Race (vs. Other Races)**

**
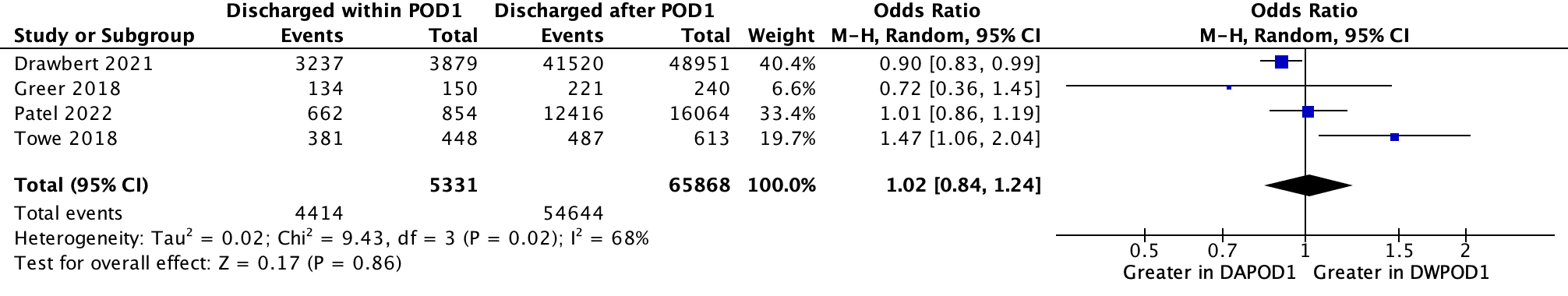
**

**Supplementary Figure 4.4: Malignant (vs. Benign) Lung Pathology**

**
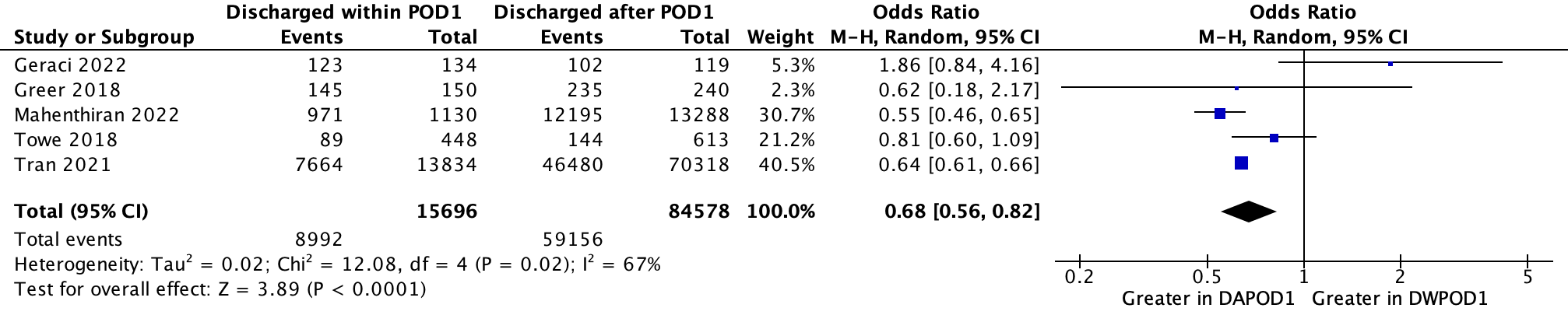
**

**Supplementary Figure 4.5: Peripheral Vascular Disease**

**
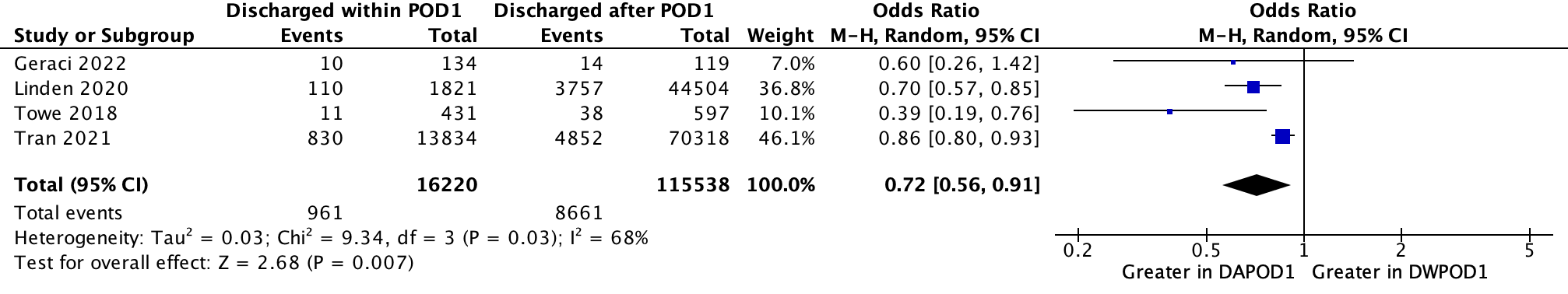
**

**Supplementary Figure 4.6: Body Mass Index (kg/m^2^)**

**
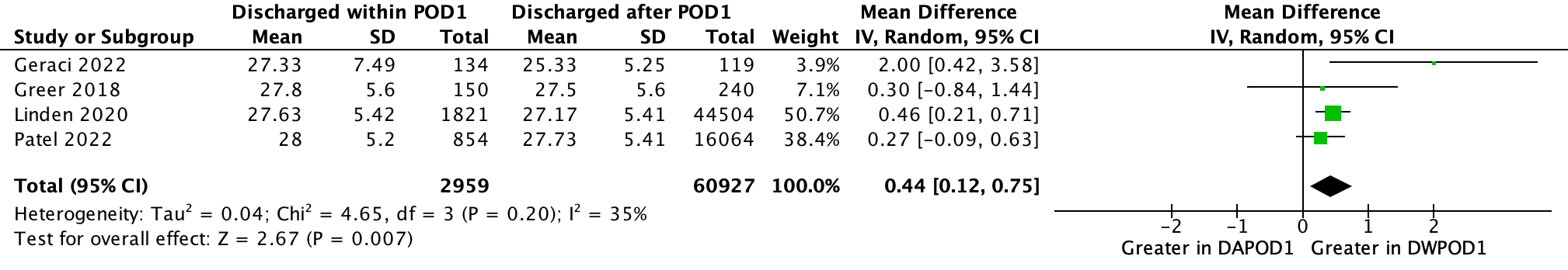
**

**Supplementary Figure 4.7: Diffusing Capacity of the Lungs for Carbon Monoxide (% of Predicted)**

**
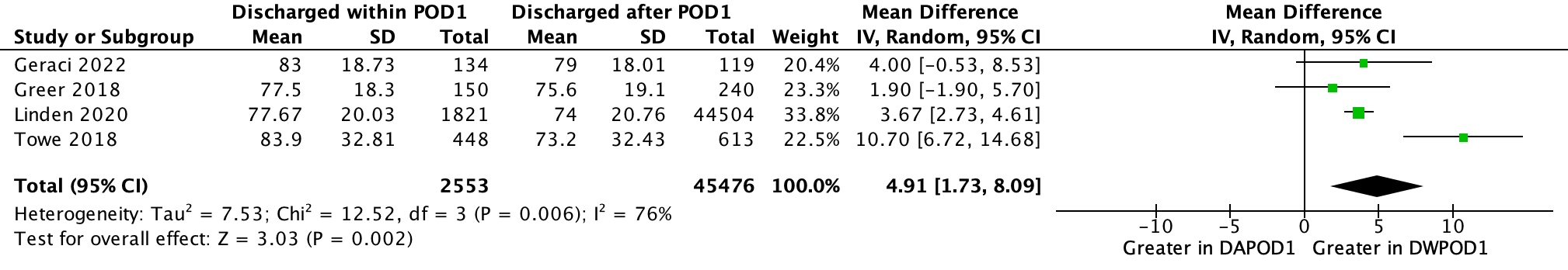
**

**Supplementary Figure 4.8: American Society of Anesthesiology Physical Status Classification <3 (vs. ASA Score ≥3)**

**
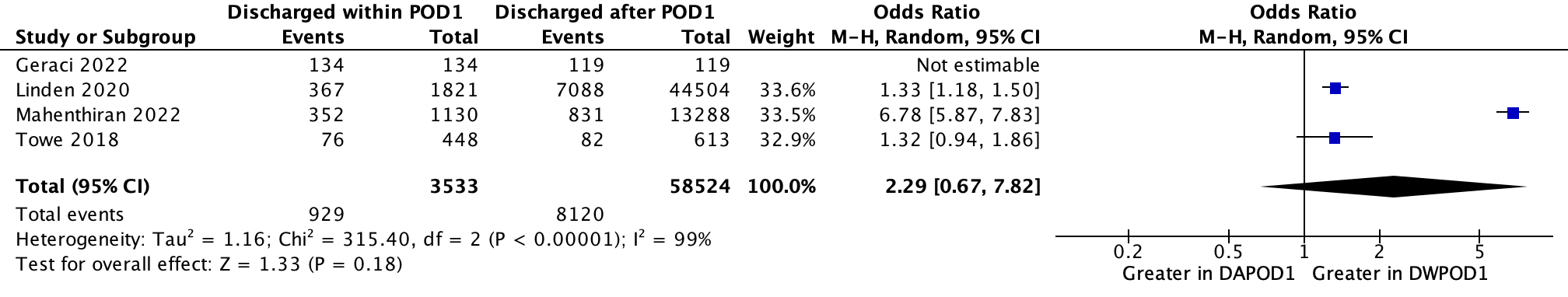
**

**Supplementary Figure 4.9: Diabetes Mellitus**

**
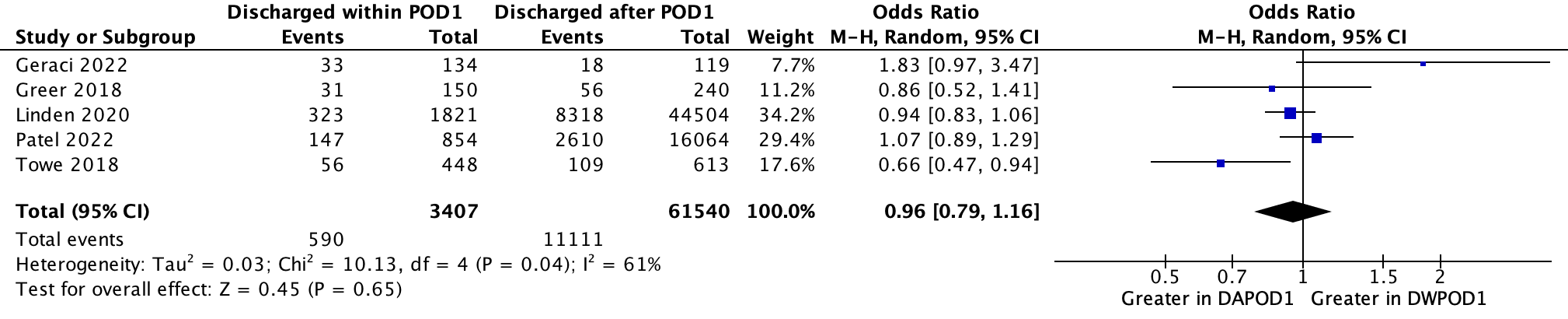
**

**Supplementary Figure 4.10: Preoperative Steroid Medications**

**
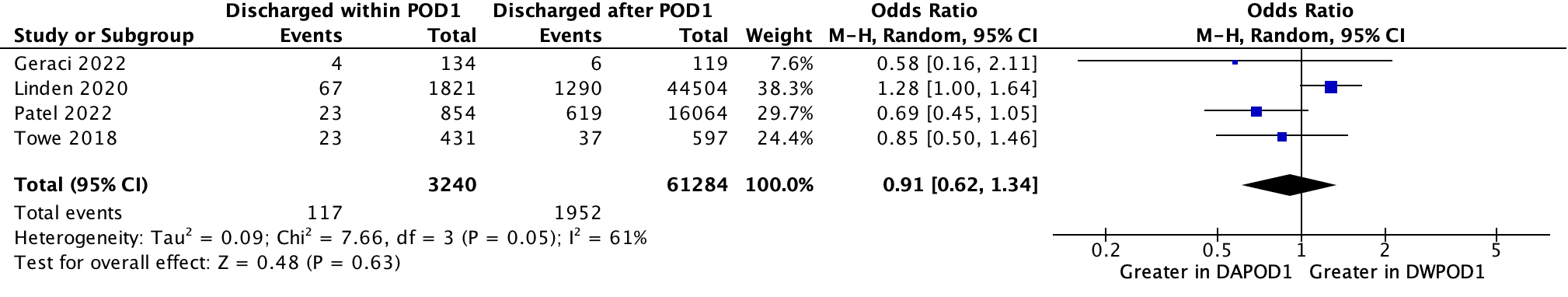
**

**Supplementary Figure 4.11: Discharged with a Chest Tube In Place**

**
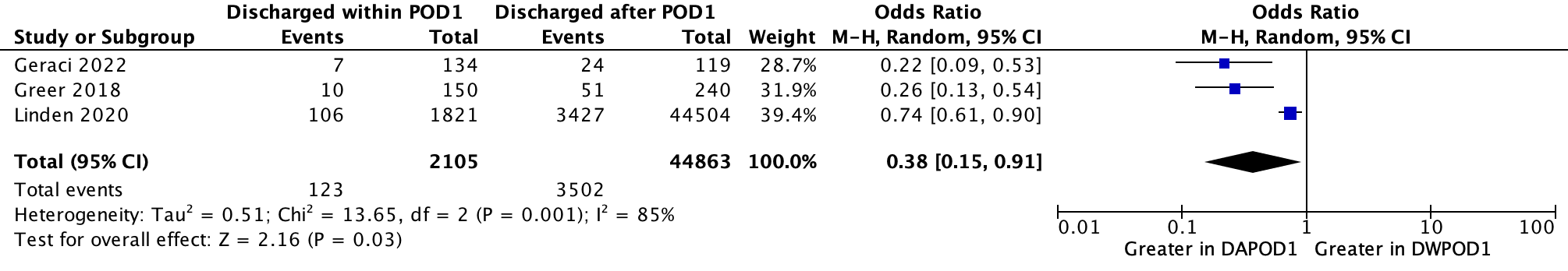
**

**Supplementary Figure 4.12: Air Leak >5 Days**

**
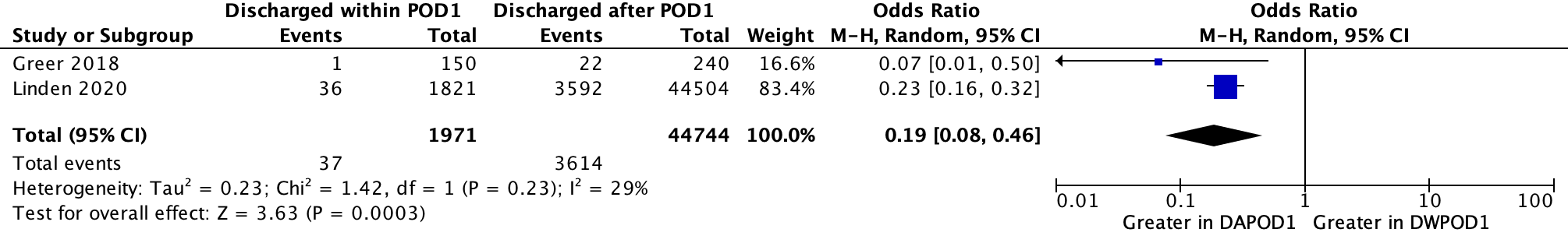
**

**Section 5: Chest Tube Removal Strategies**

**Supplementary Table:** Chest Tube Removal Strategies

| **Study** | **Chest Tube Removal Strategy** |
| --- | --- |
| **Geraci et al. (2022)** | ● If there is no air leak with a fully expanded lung or fixed pleural space deficit on the postoperative chest radiograph, the chest tube was removed on the day of surgery regardless of fluid output.  ● Patients with an air leak were discharged with the chest tube when all other discharge criteria were satisfied.  ● When the air leak resolved (0 mL/min), the patient returned to the outpatient clinic for chest tube removal. |
| **Greer et al. (2018)** | ● If no air leak is present and the total chest tube output is less than 450 mL, the tube is removed.  ● If the patient has a persistent air leak, but is otherwise medically ready for discharge, they are sent home with the chest tube to water seal.  ● If a pneumothorax causing dyspnea or significant subcutaneous emphysema occurs, the chest tube is returned to 10 cm of suction.  ● These patients are seen in the office within 72 hours of discharge for reevaluation of their air leak. If the leak is resolved, the chest tube is removed. Otherwise, the patient will be seen at 72-hour intervals for reevaluation until the chest tube is removed. |
| **Towe et al. (2018)** | ● Chest tubes were removed when there was no evidence of air leak and drainage from the tube was not sanguineous.  ● There was no formal protocol regarding an absolute volume of chest tube drainage necessary for chest tube removal. |

**Section 6: Heterogeneity of Significant Outcomes**

**Supplementary Table:** Measures of heterogeneity across all variables reaching statistical significance on meta-analysis

| **Variable** | **I ^2^** | **Tau ^2^** | **Interpretation** |
| --- | --- | --- | --- |
| Age | 99% | 3.77 | Substantial effect-size variation attributable to inter-study heterogeneity. |
| Nature of Pathology  (Malignant vs. Benign) | 70% | 0.03 | Moderate effect-size variation attributable to inter-study heterogeneity. |
| Hypertension | 74% | 0.01 | Substantial effect-size variation attributable to inter-study heterogeneity. |
| Congestive Heart Failure | 0% | 0.00 | Negligible effect-size variation attributable to inter-study heterogeneity. |
| Chronic Obstructive Pulmonary Disease | 86% | 0.03 | Substantial effect-size variation attributable to inter-study heterogeneity. |
| Coronary Artery Disease | 0% | 0.00 | Negligible effect-size variation attributable to inter-study heterogeneity. |
| Peripheral Vascular Disease | 68% | 0.03 | Moderate effect-size variation attributable to inter-study heterogeneity. |
| Body Mass Index | 35% | 0.04 | Minimal effect-size variation attributable to inter-study heterogeneity. |
| History of Smoking | 36% | 0.00 | Minimal effect-size variation attributable to inter-study heterogeneity. |
| FEV_1_ | 78% | 7.41 | Substantial effect-size variation attributable to inter-study heterogeneity. |
| DLCO | 76% | 7.53 | Substantial effect-size variation attributable to inter-study heterogeneity. |
| Type of Resection  (Lobar vs. Sublobar) | 97% | 0.13 | Substantial effect-size variation attributable to inter-study heterogeneity. |
| Duration of Operation | 99% | 187.40 | Substantial heterogeneity attributed to inter-study differences. |
| Operative Approach  (Minimally Invasive vs. Open) | 100% | 2.06 | Substantial heterogeneity attributed to inter-study differences. |
| Major Postoperative Morbidity | 95% | 0.89 | Substantial heterogeneity attributed to inter-study differences. |

**Section 7: Sensitivity Analyses**

**Supplementary Table:** Sensitivity Analyses upon removal of Tran et al. (2021)

| **Category** | **Variable** | **Effect Size** | | **Changes in Heterogeneity (I^2^)** |
| --- | --- | --- | --- | --- |
|  |  | **Original Effect Size^+^** | **Sensitivity Analysis** |  |
| Demographics | Age (in Years) | -1.65 [-2.56, -0.75] | -1.43 [-2.64, -0.22] | - |
|  | Biological Sex | 1.03 [0.91, 1.17] * | 1.02 [0.96, 1.08] * | Decreased by 80% |
| Primary Diagnosis, Comorbid Conditions and Functional Status | CHF | 0.74 [0.67, 0.81] | 0.73 [0.55, 0.98] | - |
|  | COPD | 0.70 [0.58, 0.84] | 0.76 [0.69, 0.83] | Decreased by 86% |
|  | CAD | 0.72 [0.69, 0.76] | 0.74 [0.66, 0.83] | - |
|  | HTN | 0.82 [0.73, 0.93] | 0.86 [0.80, 0.93] | - |
|  | **Indication for Cancer** | **0.68 [0.56, 0.82]** | **0.78 [0.51, 1.21] *** | **Increased by 8%** |
|  | PVD | 0.72 [0.56, 0.91] | 0.62 [0.44, 0.85] | Decreased by 41% |
|  | Smoker | 0.68 [0.61, 0.76] | 0.73 [0.54, 0.99] | Increased by 10% |
| Operative Characteristics | Lobar Resection | 0.35 [0.24, 0.51] | 0.39 [0.26, 0.57] | Decreased by 5% |
|  | MIS | 6.17 [1.91, 19.93] | 5.62 [1.73, 18.26] | Decreased by 1% |
| Postoperative Characteristics | 30-Day Mortality | 1.01 [0.50, 2.05] * | 1.23 [0.63, 2.41] * | Decreased by 33% |
|  | 30-Day Readmission | 0.84 [0.62, 1.14] * | 0.90 [0.64, 1.27] * | Decreased by 10% |
| **Results are non-significant.*  *^++^The effect size is an RR for the postoperative characteristics, MD for Age, and an OR for the remaining variables*  *CHF: Congestive Heart Failure; COPD: Chronic Obstructive Pulmonary Disease; CAD: Coronary Artery Disease; HTN: Hypertension; PVD: Peripheral Vascular Disease; MIS: Minimally Invasive Surgery* | | | | |

**Supplementary Table:** Sensitivity Analyses upon removal of Towe et al. (2018)

| **Category** | **Variable** | **Effect Size** | | **Changes in Heterogeneity (I^2^)** |
| --- | --- | --- | --- | --- |
|  |  | **Original Effect Size^+^** | **Sensitivity Analysis** |  |
| Demographics | Age (in Years) | -1.65 [-2.56, -0.75] | -1.47 [-2.70, -0.24] | Decreased by 1% |
|  | Biological Sex | 1.03 [0.91, 1.17]* | 1.03 [0.90, 1.18]* | Increased by 1% |
| Primary Diagnosis, Comorbid Conditions and Functional Status | ASA Score <3 | 2.29 [0.67, 7.82]* | 3.00 [0.60, 15.10]* | Increased by 1% |
|  | CAD | 0.72 [0.69, 0.76] | 0.72 [0.69, 0.76] | - |
|  | CHF | 0.74 [0.67, 0.81] | 0.74 [0.67, 0.81] | - |
|  | Diabetes | 0.96 [0.79, 1.16]* | 1.02 [0.86, 1.21]* | Decreased by 17% |
|  | DLCO (% of Predicted) | 4.91 [1.73, 8.09] | 3.58 [2.69, 4.48] | Decreased by 76% |
|  | FEV1 (% of Predicted) | 4.72 [1.58, 7.85] | 3.34 [2.50, 4.17] | Decreased by 78% |
|  | HTN | 0.82 [0.73, 0.93] | 0.82 [0.73, 0.93] | Increased by 5% |
|  | Indication for Cancer | 0.68 [0.56, 0.82] | 0.65 [0.51, 0.81] | Increased by 2% |
|  | Preoperative Steroids | 0.91 [0.62, 1.34]* | 0.90 [0.53, 1.55]* | Increased by 11% |
|  | PVD | 0.72 [0.56, 0.91] | 0.78 [0.65, 0.94] | Decreased by 13% |
|  | **Race** | **1.02 [0.84, 1.24]*** | **0.92 [0.85, 1.00]** | **Decreased by 68%** |
| Operative Characteristics | Operative Time | -28.08 [-41.65, -14.51] | -30.04 [-48.36, -11.73] | Decreased by 1% |
|  | MIS | 6.17 [1.91, 19.93] | 5.50 [1.56, 19.44] | - |
| Postoperative Characteristics | 30-Day Mortality | 1.01 [0.50, 2.05]* | 1.16 [0.56, 2.42]* | Increased by 2% |
|  | 30-Day Readmission | 0.84 [0.62, 1.14]* | 0.88 [0.63, 1.23]* | Increased by 2% |
| **Results are non-significant.*  *^+^The effect size is an RR for the postoperative characteristics, MD for Age, Operative Time, DLCO, FEV1, and an OR for the remaining variables*  *CHF: Congestive Heart Failure; COPD: Chronic Obstructive Pulmonary Disease; CAD: Coronary Artery Disease; HTN: Hypertension; PVD: Peripheral Vascular Disease; MIS: Minimally Invasive Surgery* | | | | |

**Section 8: Study Quality Assessment**

**Supplementary Table:** Quality assessment of included observational studies using the ROBINS-I tool for non-randomized studies of intervention

| Study | ROBINS-I Domain | | | | | | | |
| --- | --- | --- | --- | --- | --- | --- | --- | --- |
|  | Bias due to confounding | Bias in selection of participants into the study | Bias in classification of interventions | Bias due to deviations from intended interventions | Bias due to missing data | Bias in measurement of outcomes | Bias in selection of the reported result | **Overall Bias** |
| Drawbert et al.  (2021) | Moderate | Low | Low | Low | Low | Low | Low | Low |
| Geraci et al.  (2022) | Moderate | Low | Low | Low | Low | Low | Low | Low |
| Greer et al.  (2018) | Moderate | Low | Moderate | Low | Low | Low | Low | Moderate |
| Linden et al.  (2021) | Moderate | Low | Low | Low | Low | Low | Low | Low |
| Mahenthiran et al.  (2022) | Moderate | Low | Low | Low | Low | Low | Low | Low |
| Patel et al.  (2022) | Moderate | Moderate | Low | Low | Low | Low | Low | Moderate |
| Towe et al.  (2018) | Moderate | Moderate | Low | Low | Low | Low | Low | Moderate |
| Tran et al.  (2021) | Moderate | Low | Low | Low | Low | Low | Low | Low |

**Section 9: GRADE Evidence Profile**

| **Certainty assessment** | | | | | | | **№ of patients** | | **Effect** | | **Certainty** | **Importance** |
| --- | --- | --- | --- | --- | --- | --- | --- | --- | --- | --- | --- | --- |
| **№ of studies** | **Study design** | **Risk of bias** | **Inconsistency** | **Indirectness** | **Imprecision** | **Other considerations** | **Discharge Within POD1** | **Discharge After POD1** | **Relative (95% CI)** | **Absolute (95% CI)** |  |  |
| **Age (Years)** | | | | | | | | | | | | |
| 7 | observational studies | not serious | very serious^a^ | not serious | not serious | none | 21120 | 180809 | - | MD **2.76 lower** (4.15 lower to 1.37 lower) | ⨁⨁◯◯ Low |  |
| **ASA Score <3** | | | | | | | | | | | | |
| 4 | observational studies | not serious | very serious^a^ | not serious | serious^b^ | none | 929/3533 (26.3%) | 8120/58524 (13.9%) | **OR 2.29** (0.67 to 7.82) | **131 more per 1,000** (from 41 fewer to 419 more) | ⨁◯◯◯ Very low |  |
| **Preoperative Steroids** | | | | | | | | | | | | |
| 4 | observational studies | not serious | serious^c^ | not serious | not serious | none | 117/3240 (3.6%) | 1952/61284 (3.2%) | **OR 0.91** (0.62 to 1.34) | **3 fewer per 1,000** (from 12 fewer to 10 more) | ⨁⨁⨁◯ Moderate |  |
| **Congestive Heart Failure** | | | | | | | | | | | | |
| 5 | observational studies | not serious | not serious | not serious | not serious | none | 454/17074 (2.7%) | 4002/131602 (3.0%) | **OR 0.74** (0.67 to 0.81) | **8 fewer per 1,000** (from 10 fewer to 6 fewer) | ⨁⨁⨁⨁ High |  |
| **Malignant (vs. Benign) Lung Pathology** | | | | | | | | | | | | |
| 5 | observational studies | not serious | serious^c^ | not serious | not serious | strong association | 8992/15696 (57.3%) | 59156/84578 (69.9%) | **OR 0.68** (0.56 to 0.82) | **87 fewer per 1,000** (from 134 fewer to 43 fewer) | ⨁⨁⨁⨁ High |  |
| **COPD** | | | | | | | | | | | | |
| 5 | observational studies | not serious | very serious^a^ | not serious | not serious | strong association | 3726/16793 (22.2%) | 41550/131245 (31.7%) | **OR 0.70** (0.58 to 0.84) | **72 fewer per 1,000** (from 105 fewer to 36 fewer) | ⨁⨁⨁◯ Moderate |  |
| **BMI** | | | | | | | | | | | | |
| 4 | observational studies | not serious | not serious | not serious | not serious | none | 2959 | 60927 | - | MD **0.44 higher** (0.12 higher to 0.75 higher) | ⨁⨁⨁⨁ High |  |
| **Race (Caucasian vs. Other Races)** | | | | | | | | | | | | |
| 4 | observational studies | not serious | serious^c^ | not serious | not serious | none | 4414/5331 (82.8%) | 54644/65868 (83.0%) | **OR 1.02** (0.84 to 1.24) | **3 more per 1,000** (from 26 fewer to 28 more) | ⨁⨁⨁◯ Moderate |  |
| **Hypertension** | | | | | | | | | | | | |
| 6 | observational studies | not serious | serious^c^ | not serious | not serious | none | 8930/17224 (51.8%) | 78357/131842 (59.4%) | **OR 0.82** (0.73 to 0.93) | **49 fewer per 1,000** (from 78 fewer to 18 fewer) | ⨁⨁⨁◯ Moderate |  |
| **Diabetes** | | | | | | | | | | | | |
| 5 | observational studies | not serious | serious^c^ | not serious | not serious | none | 590/3407 (17.3%) | 11111/61540 (18.1%) | **OR 0.96** (0.79 to 1.16) | **6 fewer per 1,000** (from 32 fewer to 23 more) | ⨁⨁⨁◯ Moderate |  |
| **Coronary Artery Disease** | | | | | | | | | | | | |
| 5 | observational studies | not serious | not serious | not serious | not serious | none | 2117/16370 (12.9%) | 20885/115778 (18.0%) | **OR 0.72** (0.69 to 0.76) | **44 fewer per 1,000** (from 49 fewer to 37 fewer) | ⨁⨁⨁⨁ High |  |
| **Peripheral Vascular Disease** | | | | | | | | | | | | |
| 4 | observational studies | not serious | serious^c^ | not serious | not serious | none | 961/16220 (5.9%) | 8661/115538 (7.5%) | **OR 0.72** (0.56 to 0.91) | **20 fewer per 1,000** (from 32 fewer to 6 fewer) | ⨁⨁⨁◯ Moderate |  |
| **FEV1 (% of Predicted)** | | | | | | | | | | | | |
| 4 | observational studies | not serious | very serious^a^ | not serious | not serious | none | 2553 | 45476 | - | MD **4.72 higher** (1.58 higher to 7.85 higher) | ⨁⨁◯◯ Low |  |
| **Smoker** | | | | | | | | | | | | |
| 4 | observational studies | not serious | not serious | not serious | not serious | strong association | 7845/14972 (52.4%) | 50475/86741 (58.2%) | **OR 0.68** (0.61 to 0.76) | **96 fewer per 1,000** (from 123 fewer to 68 fewer) | ⨁⨁⨁⨁ High |  |
| **Location of Tumour (Upper Lobe vs. Lower/Middle Lobes)** | | | | | | | | | | | | |
| 4 | observational studies | not serious | very serious^a^ | not serious | not serious | none | 3181/5984 (53.2%) | 52760/93814 (56.2%) | **OR 0.84** (0.67 to 1.04) | **43 fewer per 1,000** (from 100 fewer to 10 more) | ⨁⨁◯◯ Low |  |
| **Biological Sex (Females vs. Males)** | | | | | | | | | | | | |
| 7 | observational studies | not serious | very serious^a^ | not serious | not serious | none | 9490/21120 (44.9%) | 74809/180809 (41.4%) | **OR 1.03** (0.91 to 1.17) | **7 more per 1,000** (from 23 fewer to 39 more) | ⨁⨁◯◯ Low |  |
| **DLCO (% of Predicted)** | | | | | | | | | | | | |
| 4 | observational studies | not serious | serious^c^ | not serious | not serious | none | 2553 | 45476 | - | MD **4.91 higher** (1.73 higher to 8.09 higher) | ⨁⨁⨁◯ Moderate |  |

**CI:** confidence interval; **MD:** mean difference; **OR:** odds ratio

| **Certainty assessment** | | | | | | | **№ of patients** | | **Effect** | | **Certainty** | **Importance** |
| --- | --- | --- | --- | --- | --- | --- | --- | --- | --- | --- | --- | --- |
| **№ of studies** | **Study design** | **Risk of bias** | **Inconsistency** | **Indirectness** | **Imprecision** | **Other considerations** | **Discharge Within POD1** | **Discharge After POD1** | **Relative (95% CI)** | **Absolute (95% CI)** |  |  |
| **Operative Time (Minutes)** | | | | | | | | | | | | |
| 4 | observational studies | not serious | very serious^a^ | not serious | serious^b^ | none | 3257 | 61300 | - | MD **28.08 lower** (41.65 lower to 14.51 lower) | ⨁◯◯◯ Very low |  |
| **Minimally Invasive Surgery** | | | | | | | | | | | | |
| 7 | observational studies | not serious | very serious^a^ | not serious | serious^b^ | very strong association | 17754/21120 (84.1%) | 112024/180809 (62.0%) | **OR 6.17** (1.91 to 19.93) | **290 more per 1,000** (from 137 more to 351 more) | ⨁⨁⨁◯ Moderate |  |
| **Lobar Resection** | | | | | | | | | | | | |
| 4 | observational studies | not serious | very serious^a^ | not serious | not serious | very strong association | 5647/16904 (33.4%) | 89526/127994 (69.9%) | **OR 0.35** (0.24 to 0.51) | **251 fewer per 1,000** (from 341 fewer to 157 fewer) | ⨁⨁⨁⨁ High |  |

**CI:** confidence interval; **MD:** mean difference; **OR:** odds ratio

| **Certainty assessment** | | | | | | | **№ of patients** | | **Effect** | | **Certainty** | **Importance** |
| --- | --- | --- | --- | --- | --- | --- | --- | --- | --- | --- | --- | --- |
| **№ of studies** | **Study design** | **Risk of bias** | **Inconsistency** | **Indirectness** | **Imprecision** | **Other considerations** | **Discharge Within POD1** | **Discharge After POD1** | **Relative (95% CI)** | **Absolute (95% CI)** |  |  |
| **30- Day Mortality** | | | | | | | | | | | | |
| 7 | observational studies | not serious | very serious^a^ | not serious | not serious | none | 276/21062 (1.3%) | 1684/141346 (1.2%) | **RR 1.01** (0.50 to 2.05) | **0 fewer per 1,000** (from 6 fewer to 13 more) | ⨁⨁◯◯ Low |  |
| **30-Day Readmission** | | | | | | | | | | | | |
| 7 | observational studies | not serious | very serious^a^ | not serious | not serious | none | 836/20994 (4.0%) | 7157/120401 (5.9%) | **RR 0.84** (0.62 to 1.14) | **10 fewer per 1,000** (from 23 fewer to 8 more) | ⨁⨁◯◯ Low |  |
| **Postoperative Morbidity** | | | | | | | | | | | | |
| 3 | observational studies | not serious | not serious | not serious | not serious | strong association | 57/2825 (2.0%) | 4375/66475 (6.6%) | **RR 0.31** (0.24 to 0.41) | **45 fewer per 1,000** (from 50 fewer to 39 fewer) | ⨁⨁⨁⨁ High |  |
| **Discharged With Chest Tube** | | | | | | | | | | | | |
| 3 | observational studies | not serious | very serious^a^ | not serious | not serious | strong association | 123/2105 (5.8%) | 3502/44863 (7.8%) | **OR 0.38** (0.15 to 0.91) | **47 fewer per 1,000** (from 66 fewer to 7 fewer) | ⨁⨁⨁◯ Moderate |  |
| **Air Leak (>5 Days)** | | | | | | | | | | | | |
| 2 | observational studies | not serious | not serious | not serious | not serious | very strong association | 37/1971 (1.9%) | 3614/44744 (8.1%) | **OR 0.19** (0.08 to 0.46) | **64 fewer per 1,000** (from 74 fewer to 42 fewer) | ⨁⨁⨁⨁ High |  |

**CI:** confidence interval; **OR:** odds ratio; **RR:** risk ratio

#### Explanations

a. Substantial heterogeneity

b. Very wide confidence interval

c. Moderate heterogeneity
